# Supplementary figures and images for: Look twice: A generalist computational model predicts return fixations across tasks and species
Source: PLoS Comput Biol. 2022 Nov 22;18(11):e1010654. doi: 10.1371/journal.pcbi.1010654 (PMC9681066; doi:10.1371/journal.pcbi.1010654)

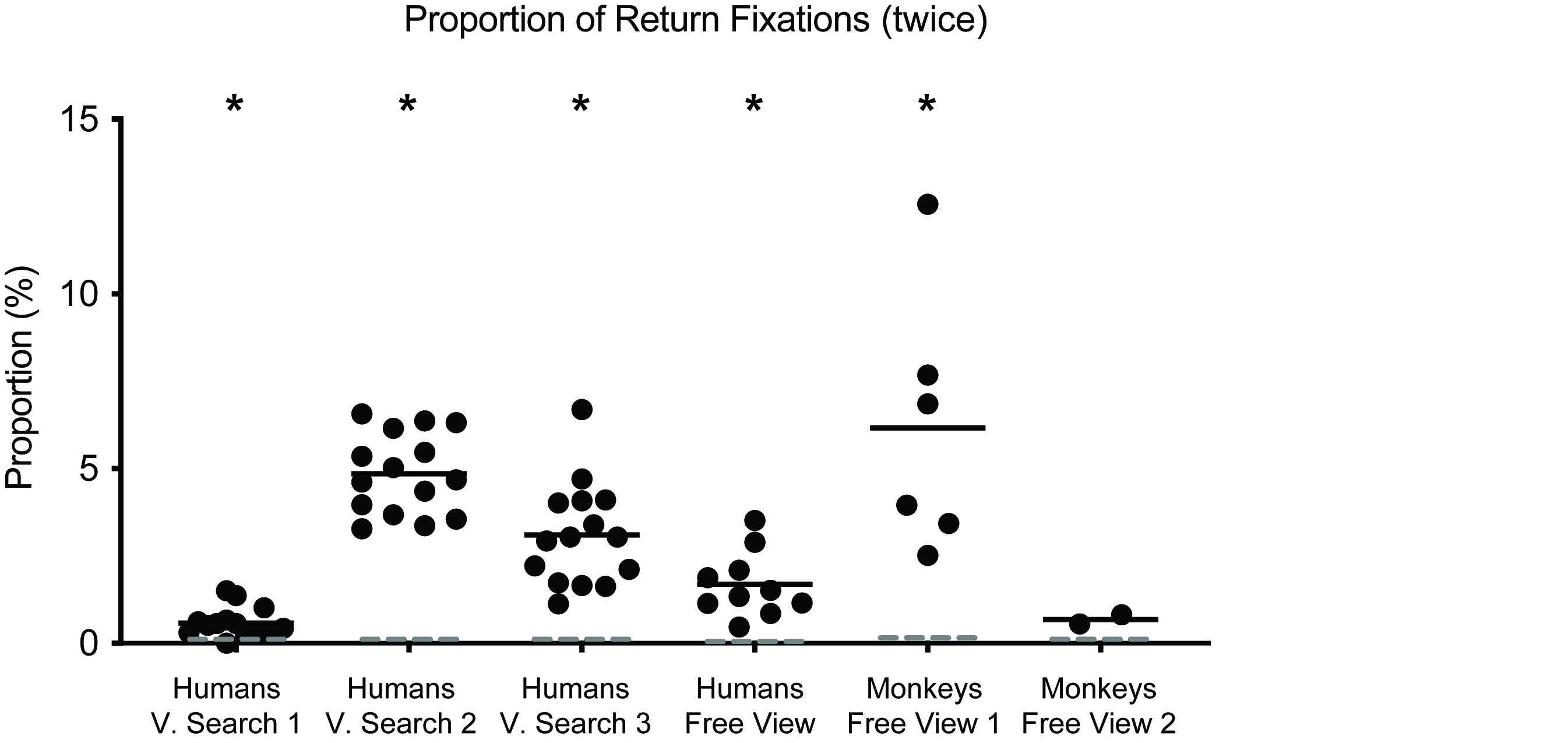

Supplement: S1 Fig — (TIF) [file pcbi.1010654.s001.tif]

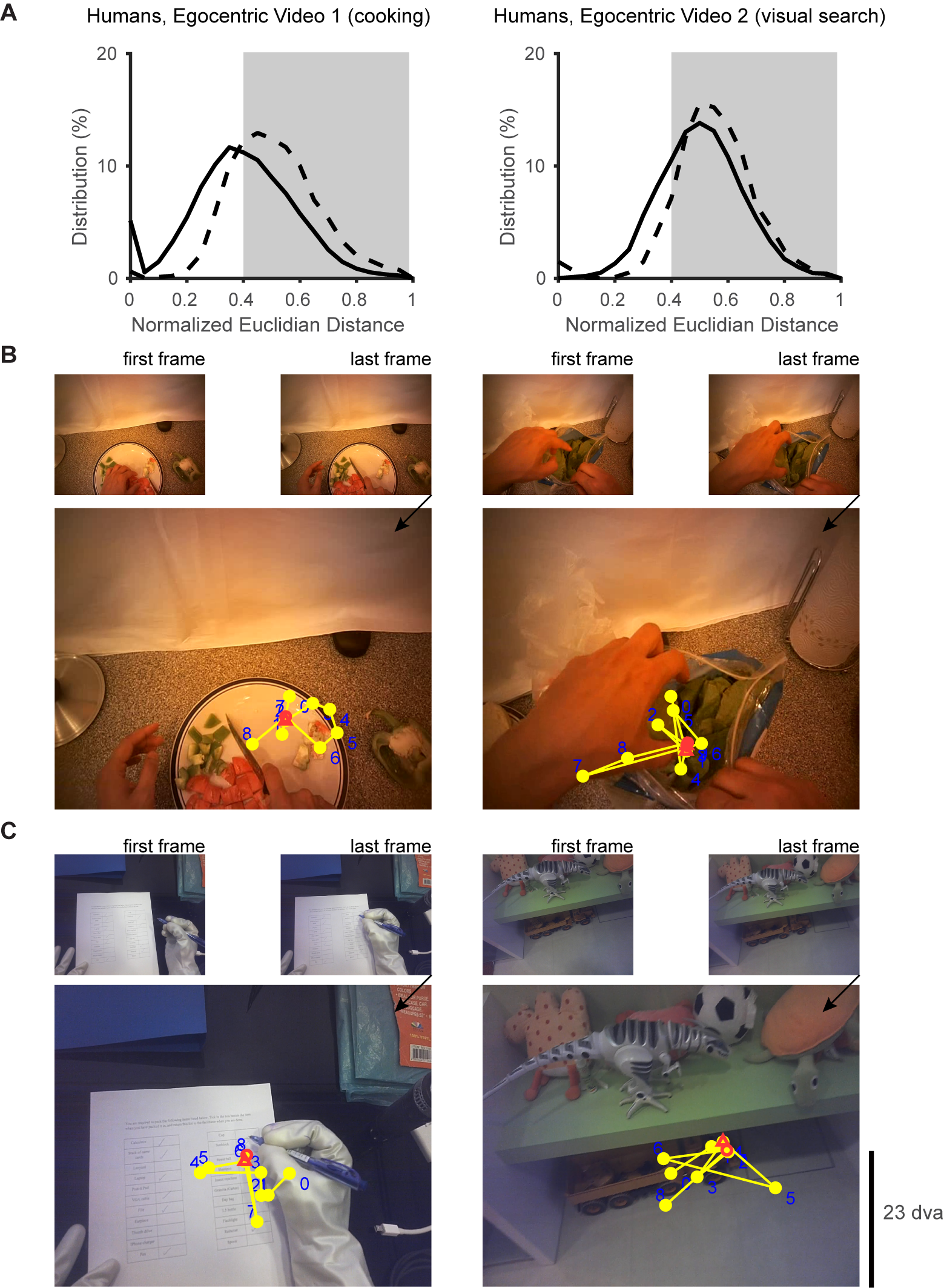

Supplement: S2 Fig — (TIF) [file pcbi.1010654.s002.tif]

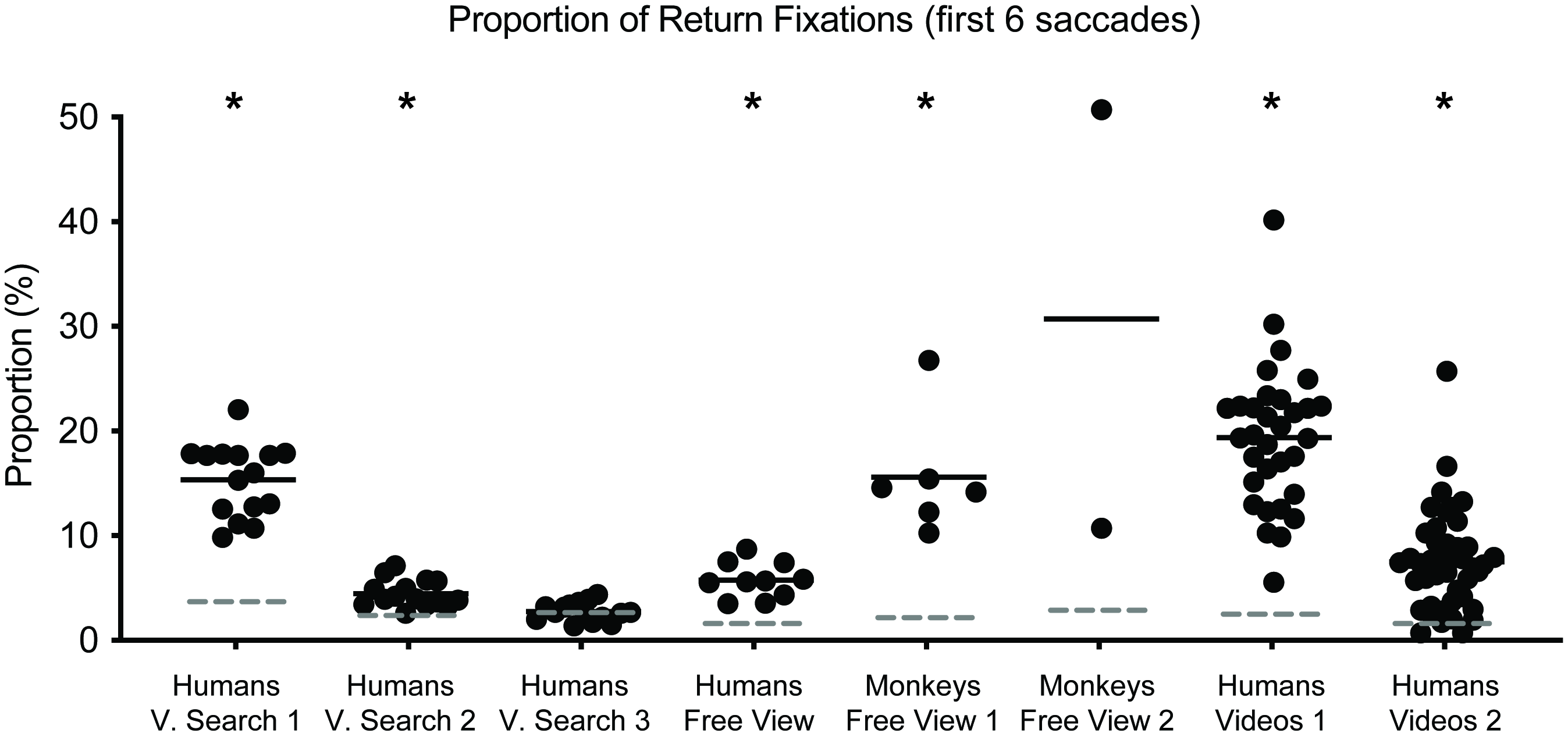

Supplement: S3 Fig — (TIF) [file pcbi.1010654.s003.tif]

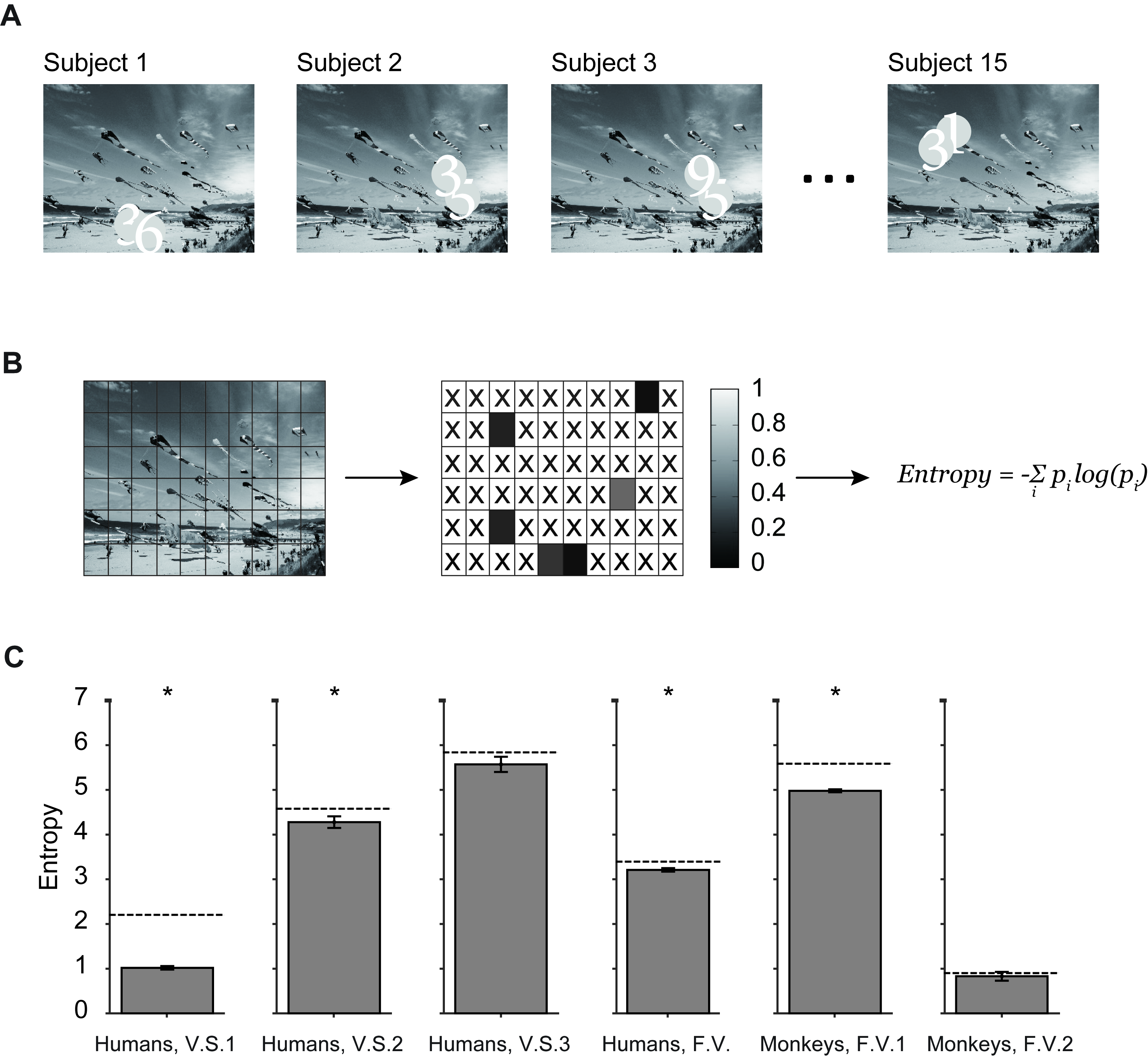

Supplement: S4 Fig — (TIF) [file pcbi.1010654.s004.tif]

**A1**

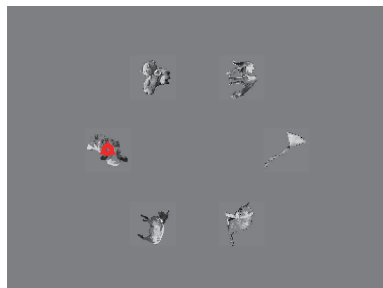

**A2**

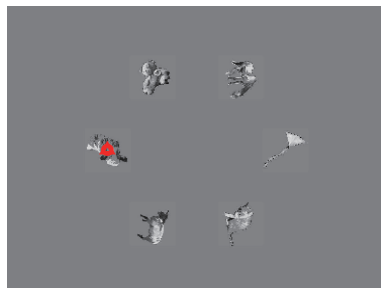

**A3**

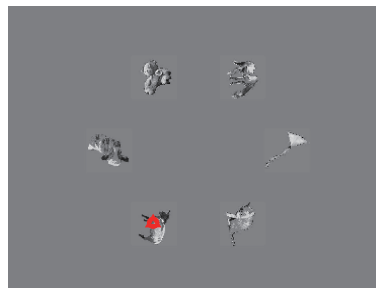

**A4**

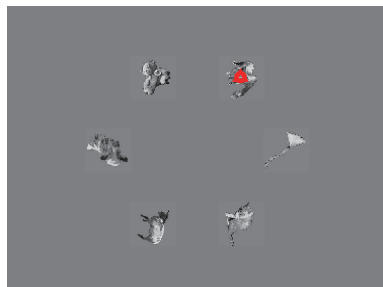

**A5**

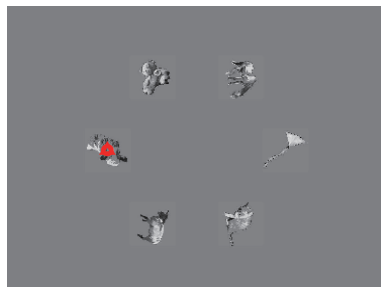

**A6**

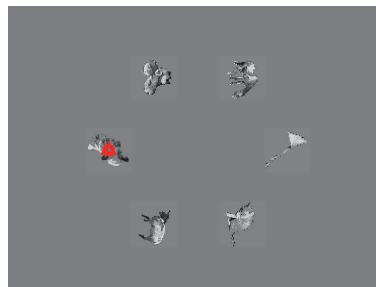

**A7**

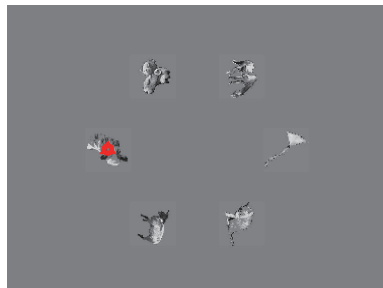

**A8**

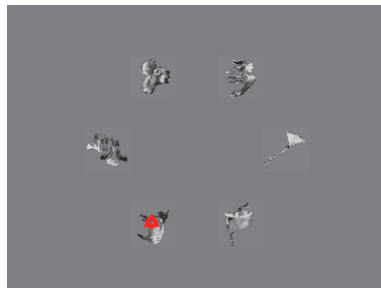

**A9**

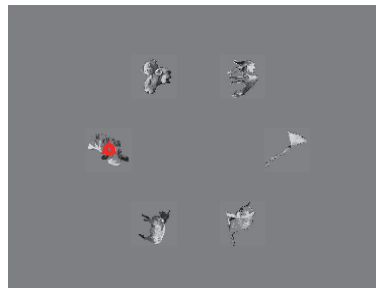

**A10**

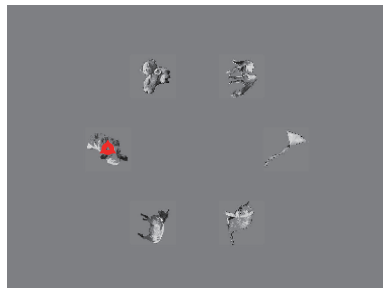

**A11**

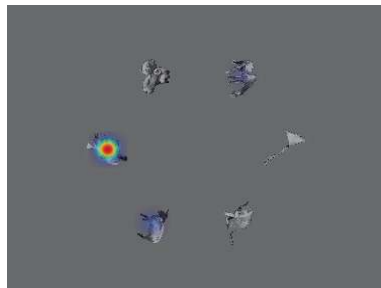

12.5 dva

**B1**

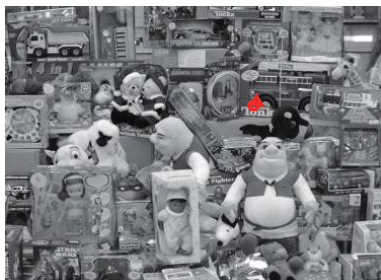

**B2**

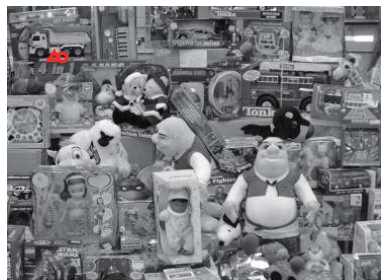

**B3**

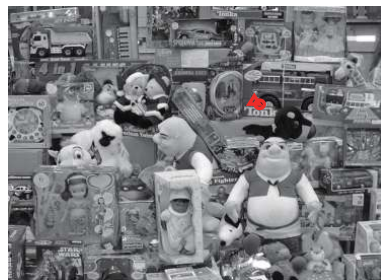

**B4**

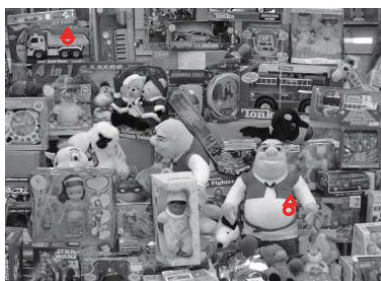

**B5**

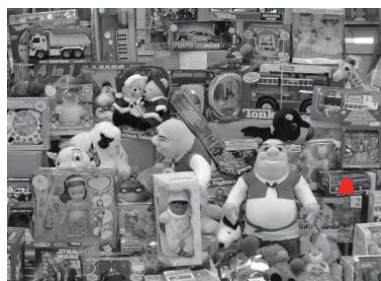

**B6**

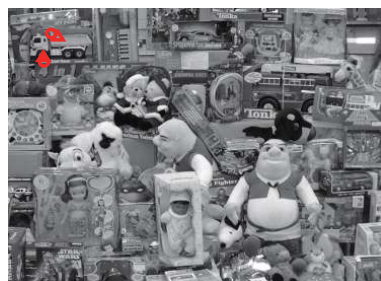

**B7**

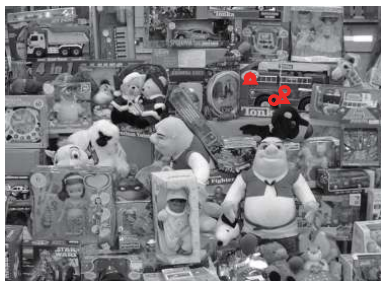

**B8**

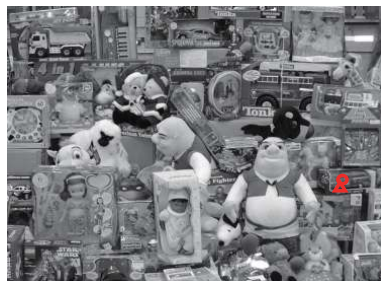

**B9**

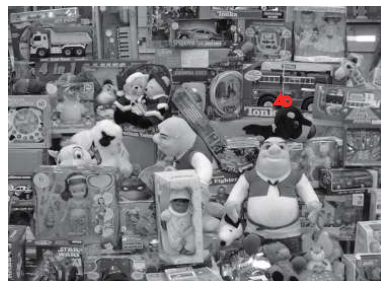

**B10**

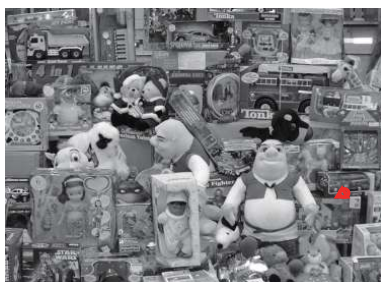

**B11**

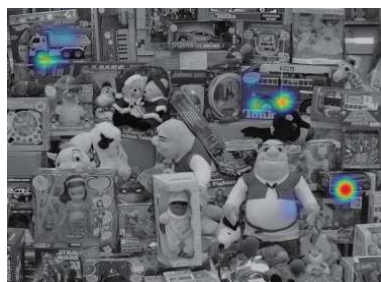

12.5 dva

C1

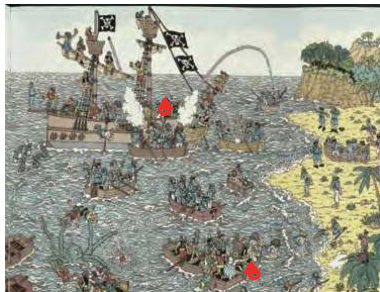

C2

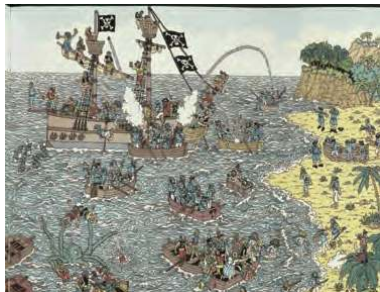

C3

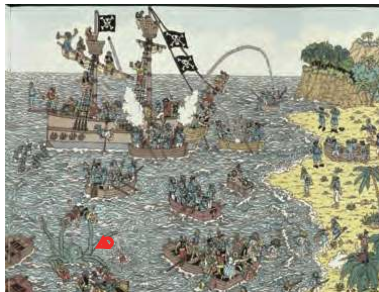

C4

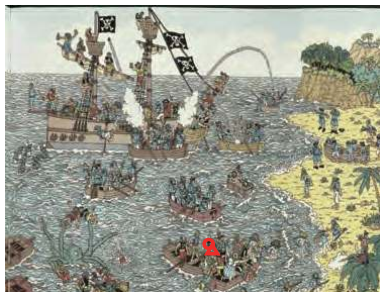

C5

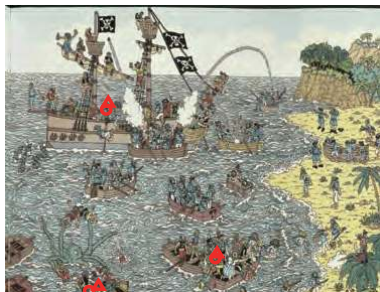

C6

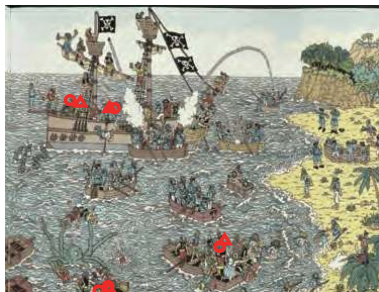

C7

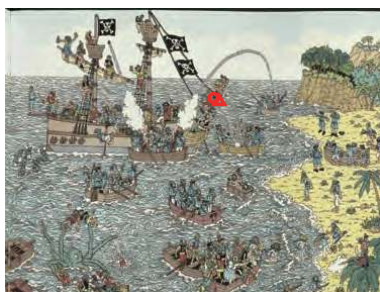

C8

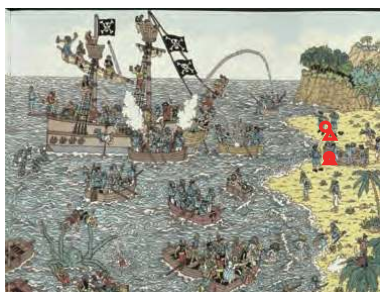

C9

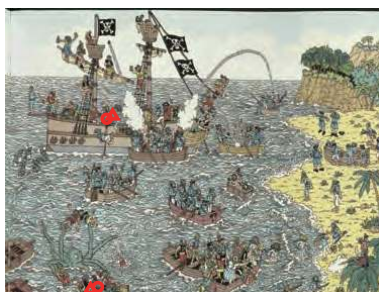

C10

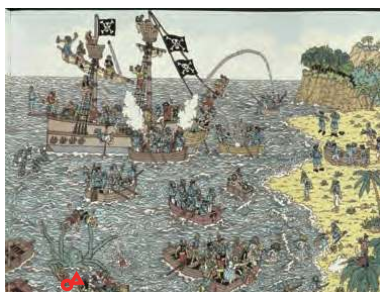

C11

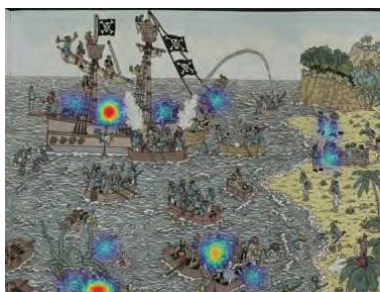

12.5 dva

D1

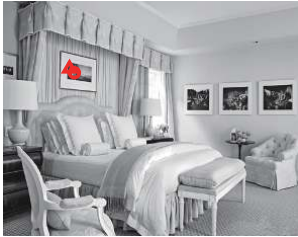

D2

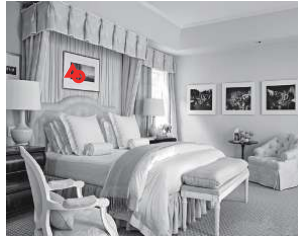

D3

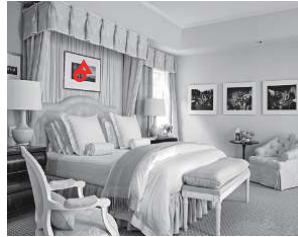

D4

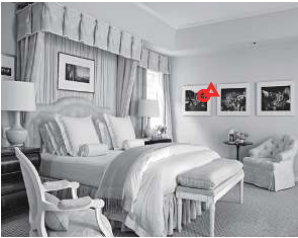

D5

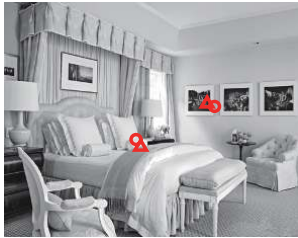

D6

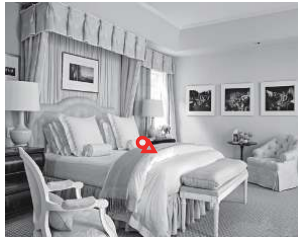

D7

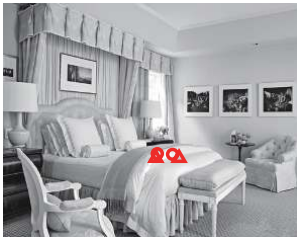

D8

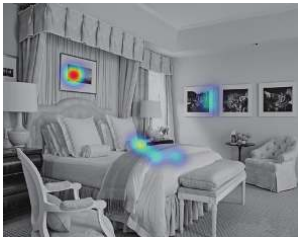

12.5 dva

E1

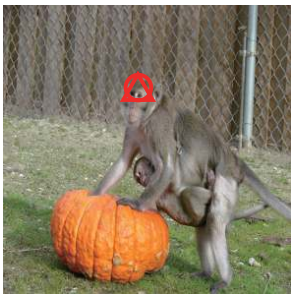

E2

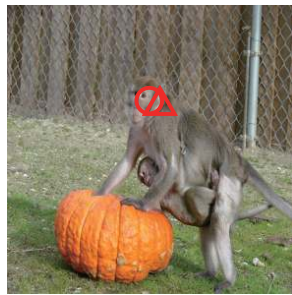

E3

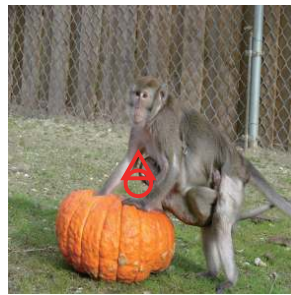

E4

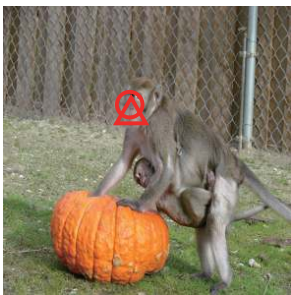

E5

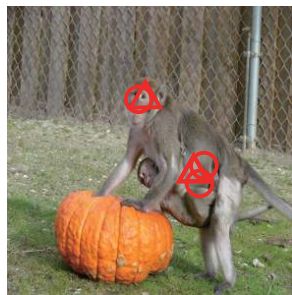

E6

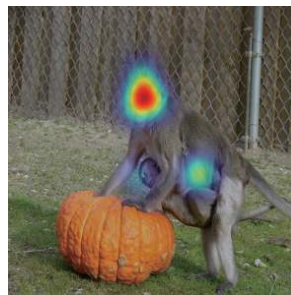

8 dva

**F1**

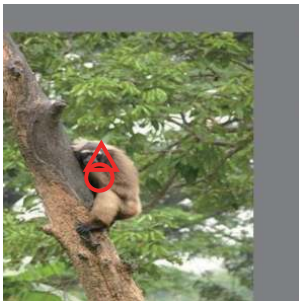

**F2**

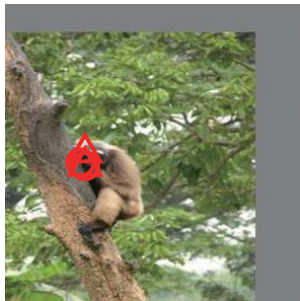

**F3**

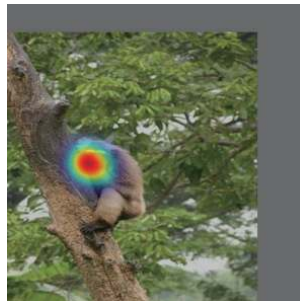

7.5 dva

Supplement: S5 Fig — (PDF) [file pcbi.1010654.s005.pdf]

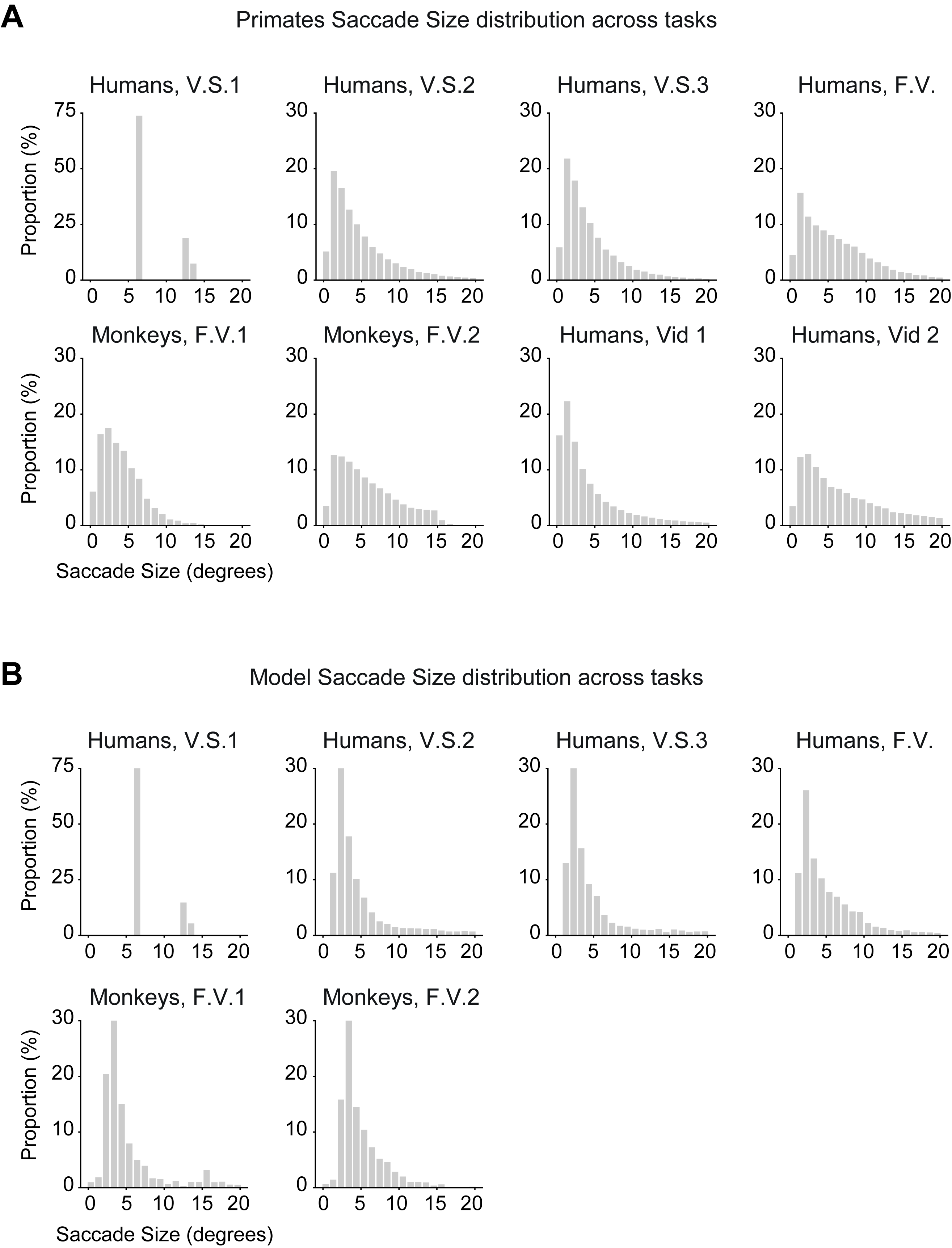

Supplement: S6 Fig — (TIF) [file pcbi.1010654.s006.tif]

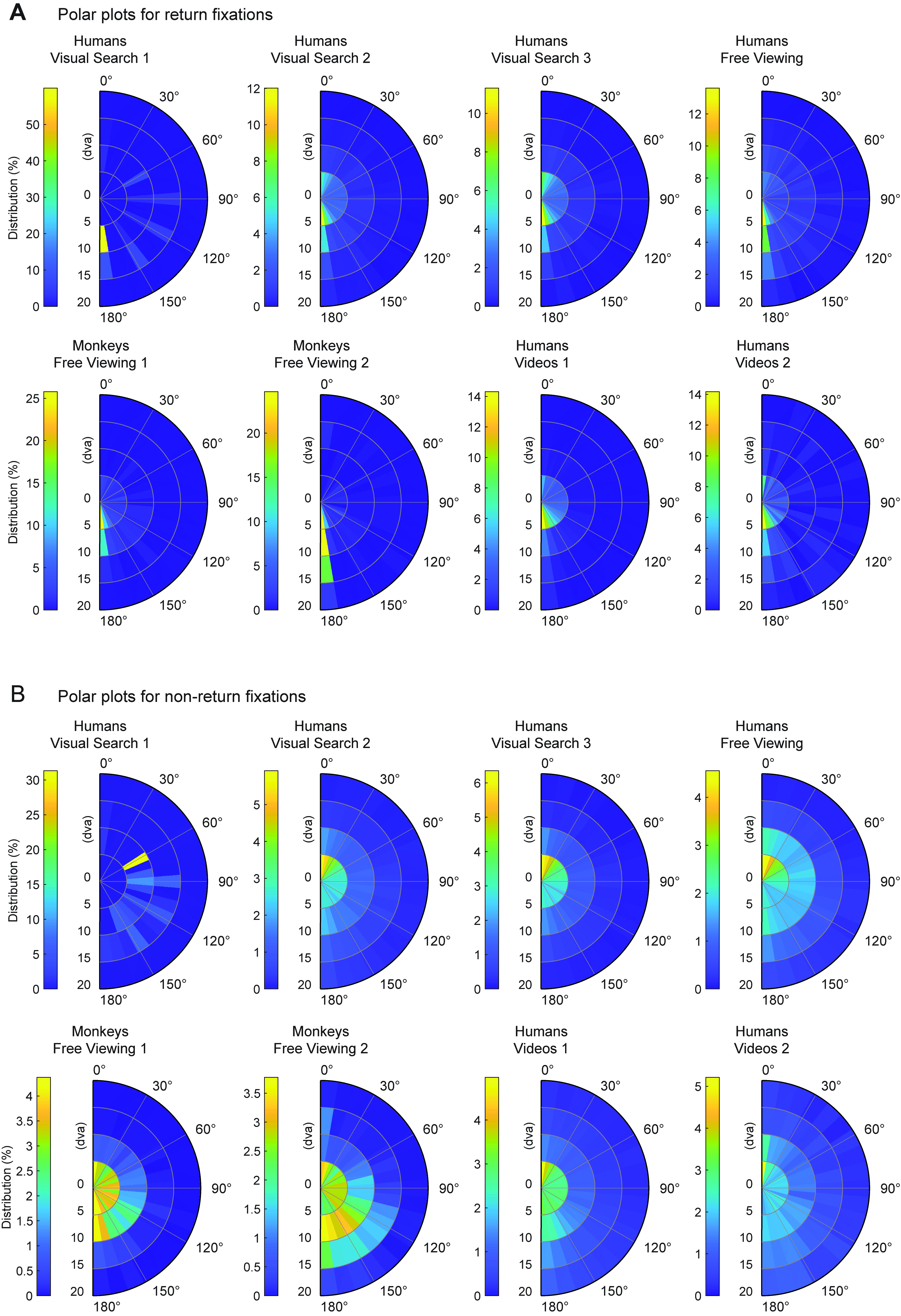

Supplement: S7 Fig — (TIF) [file pcbi.1010654.s007.tif]

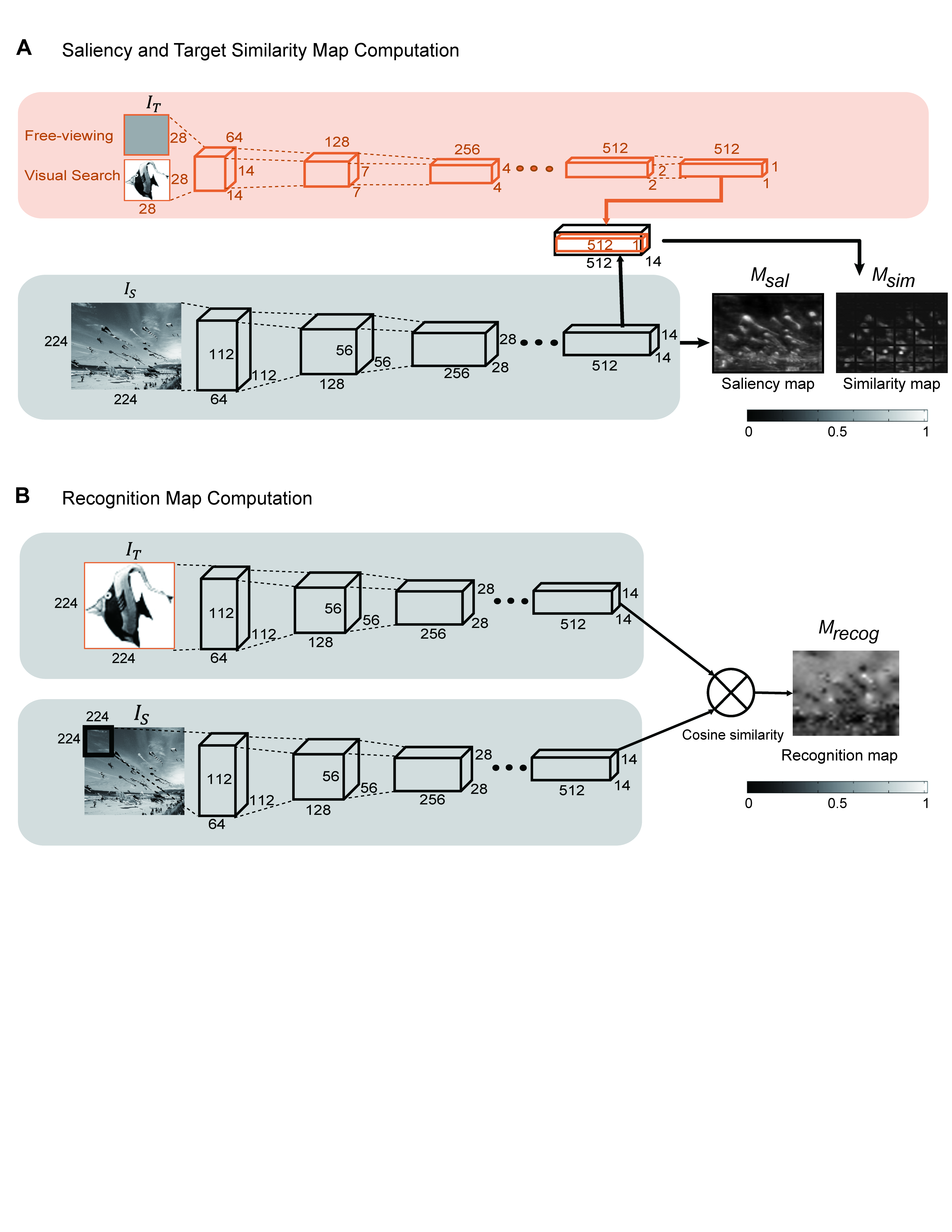

Supplement: S9 Fig — (TIF) [file pcbi.1010654.s009.tif]

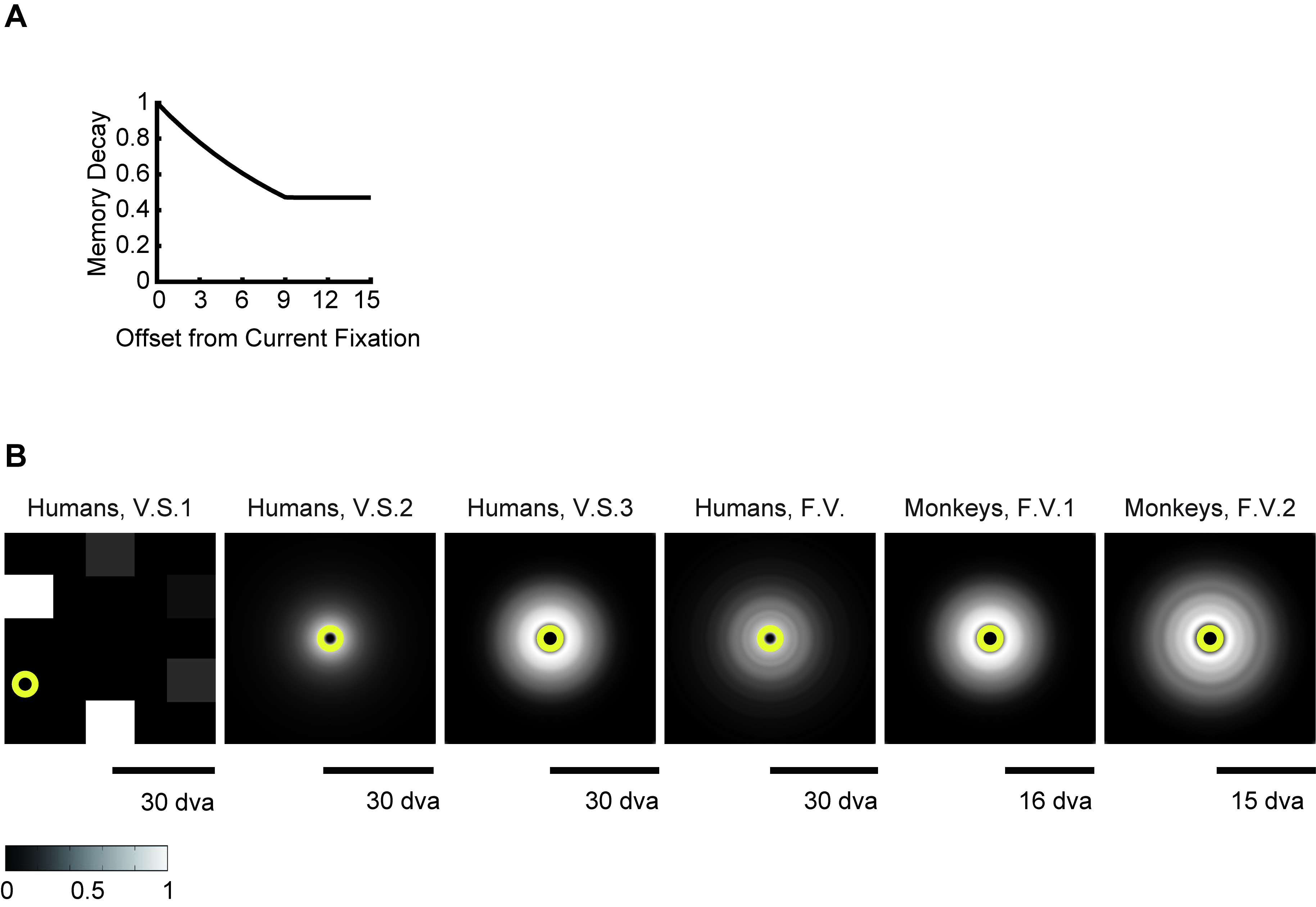

Supplement: S10 Fig — (TIF) [file pcbi.1010654.s010.tif]

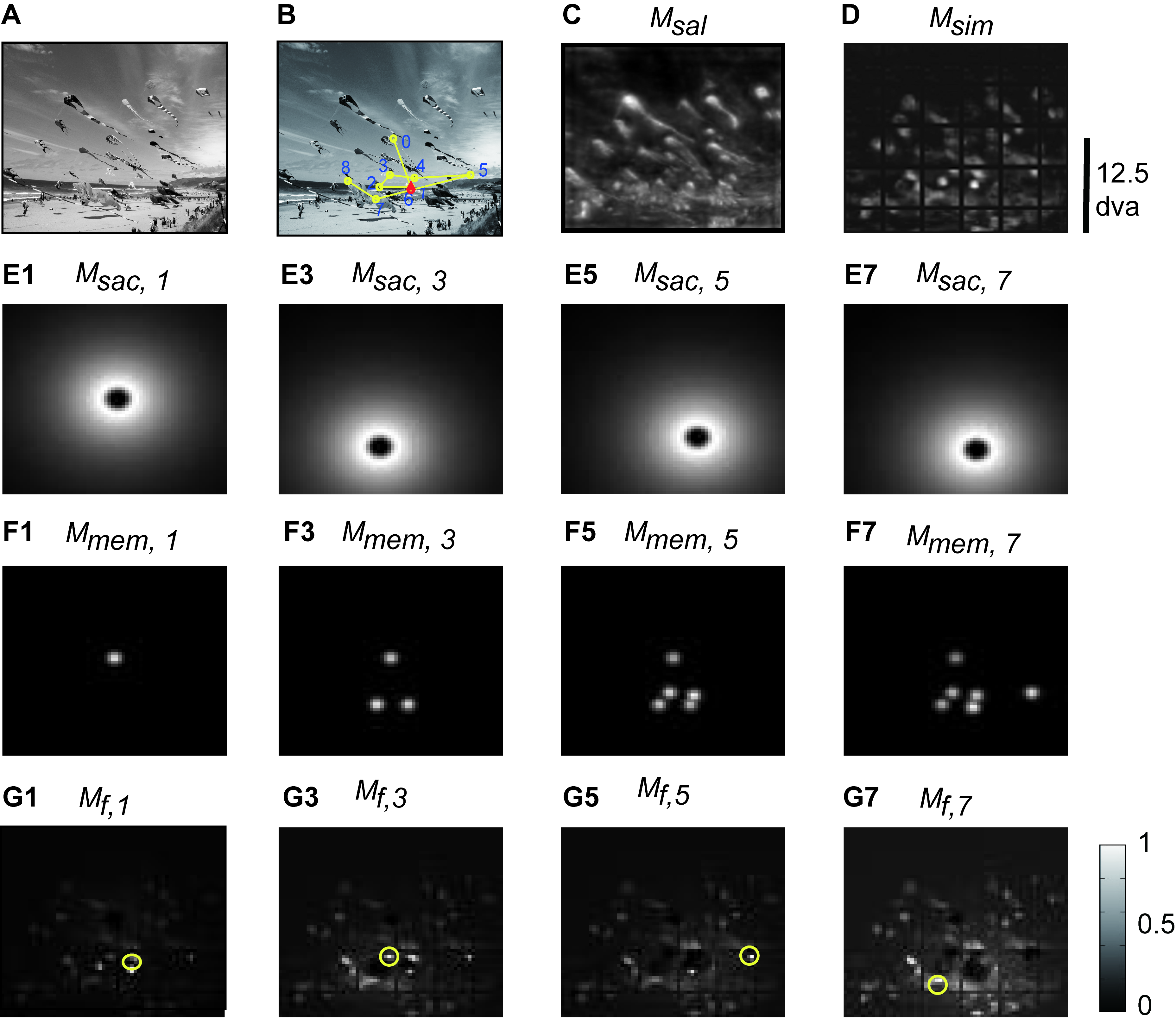

Supplement: S11 Fig — (TIF) [file pcbi.1010654.s011.tif]

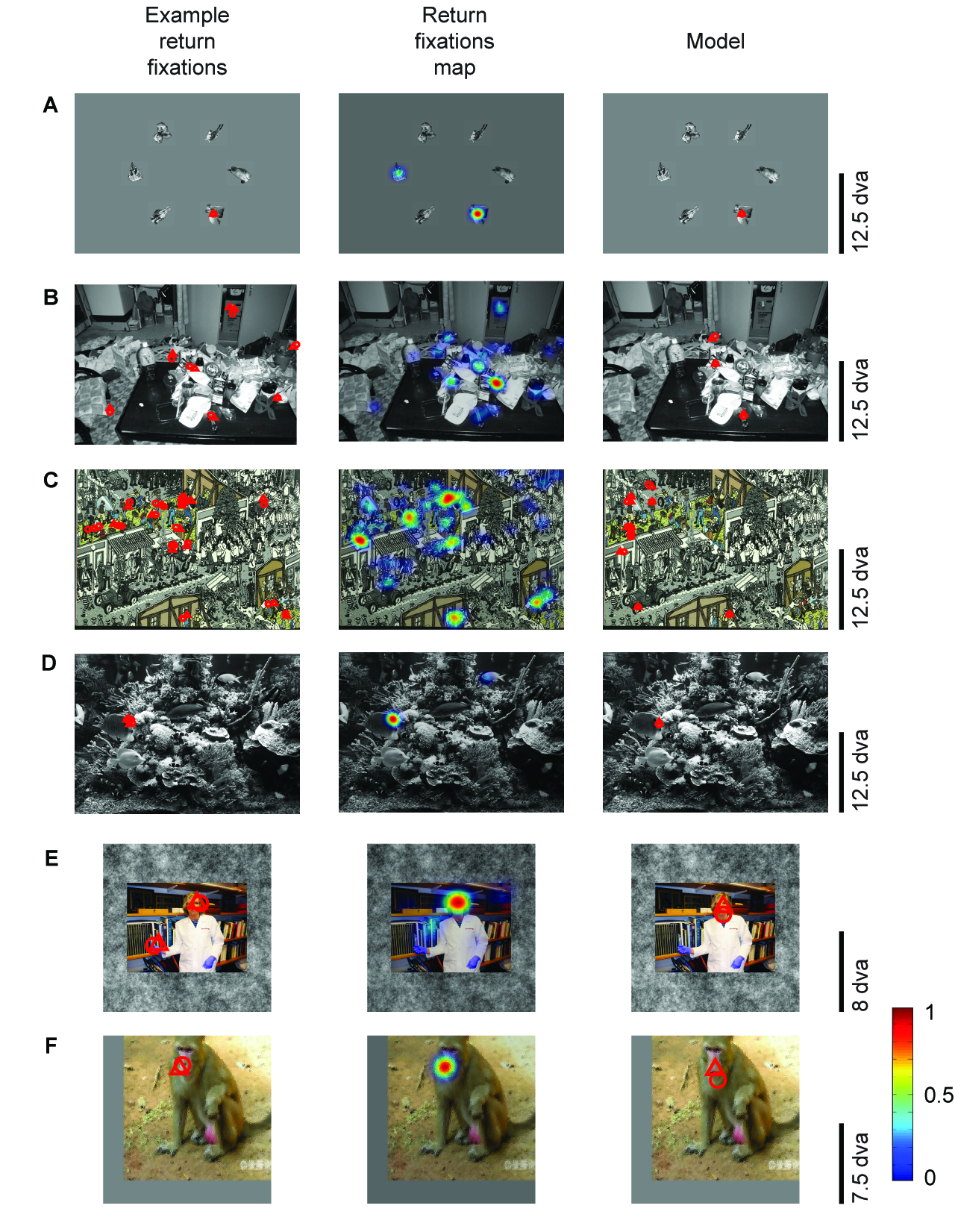

Supplement: S12 Fig — (TIF) [file pcbi.1010654.s012.tif]

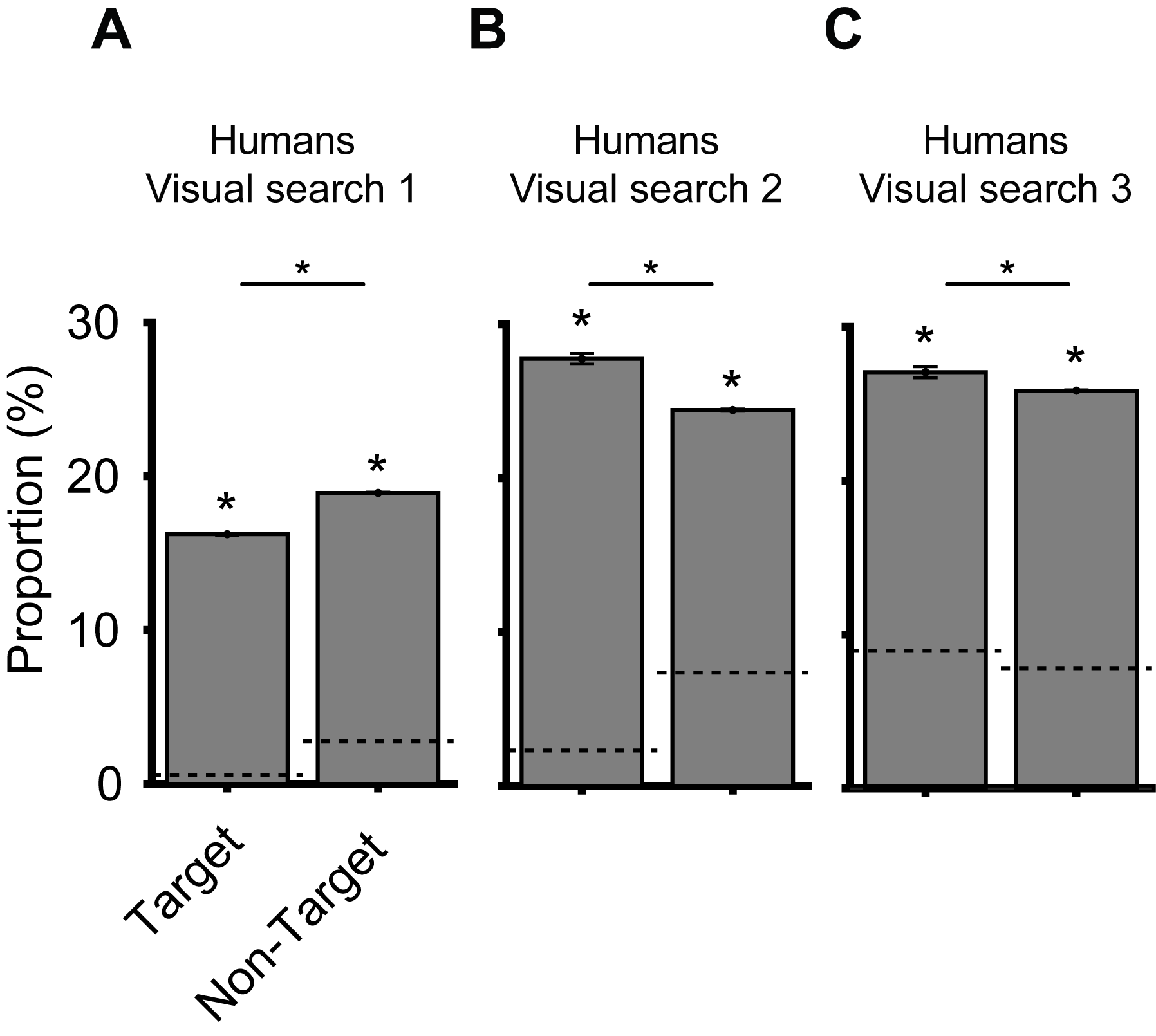

Supplement: S13 Fig — (TIF) [file pcbi.1010654.s013.tif]

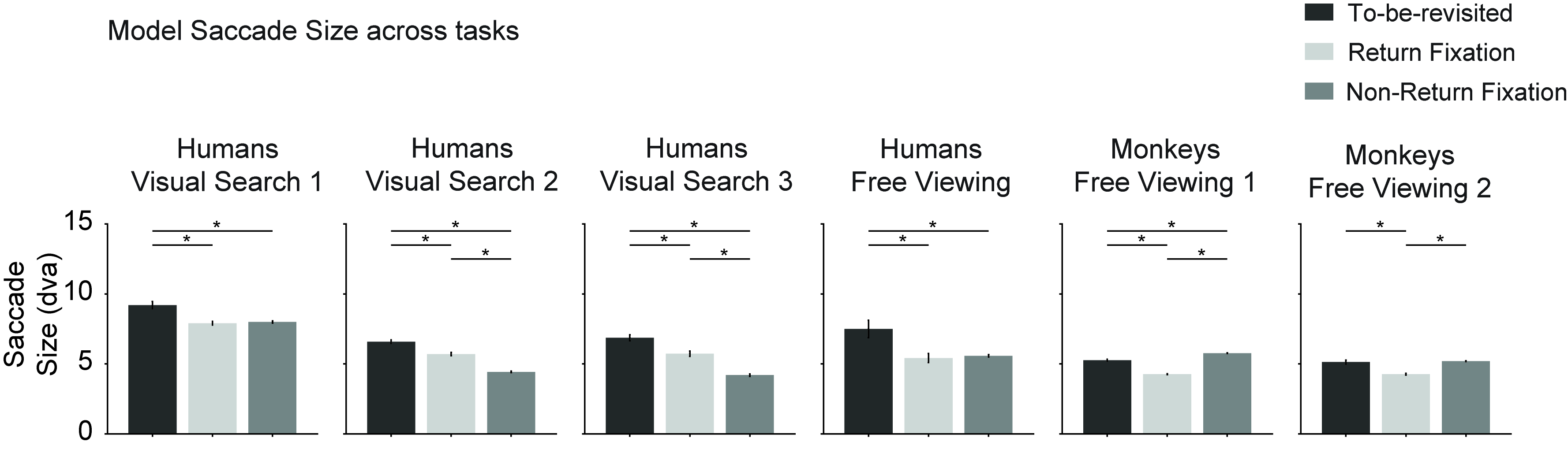

Supplement: S14 Fig — (TIF) [file pcbi.1010654.s014.tif]

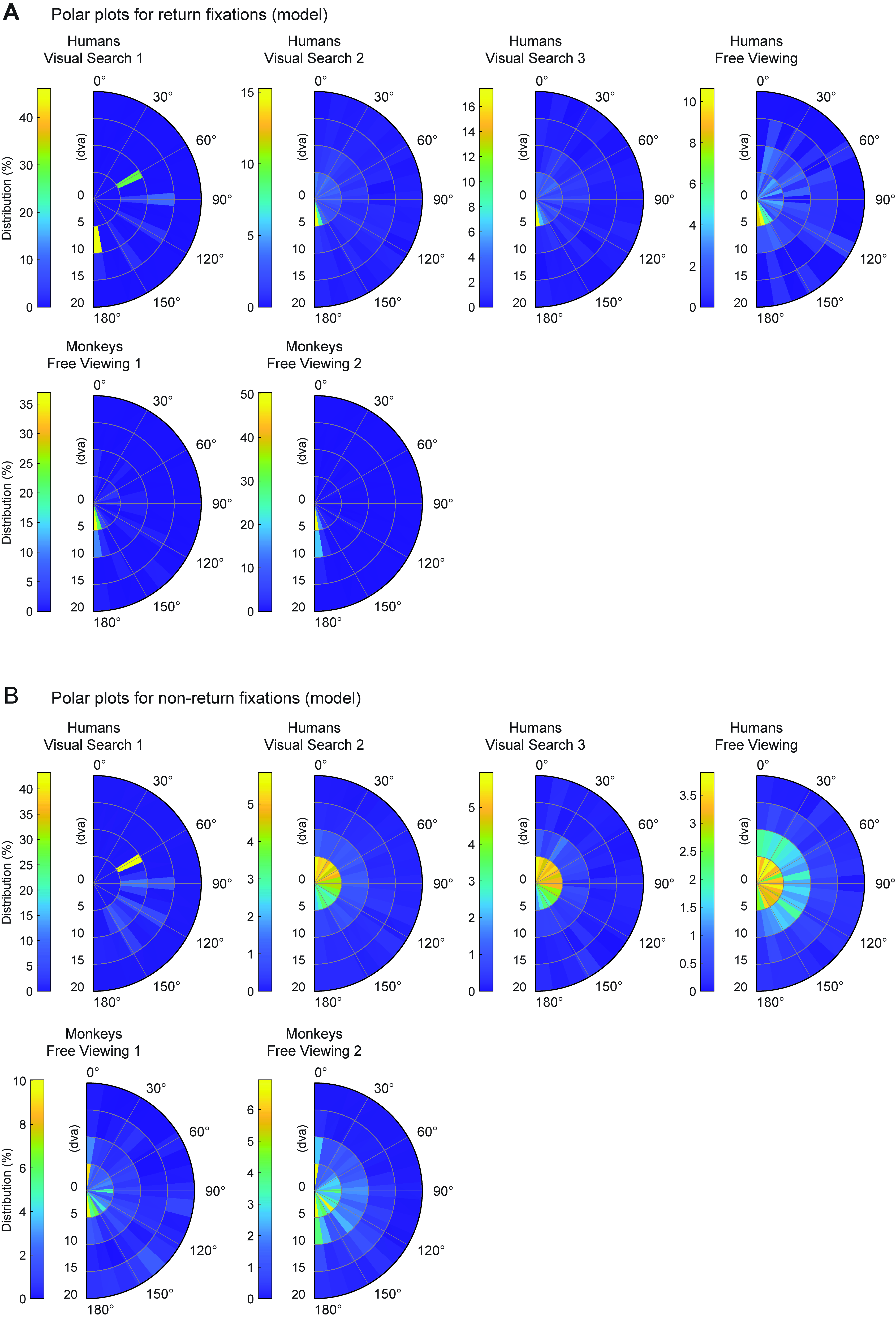

Supplement: S15 Fig — (TIF) [file pcbi.1010654.s015.tif]

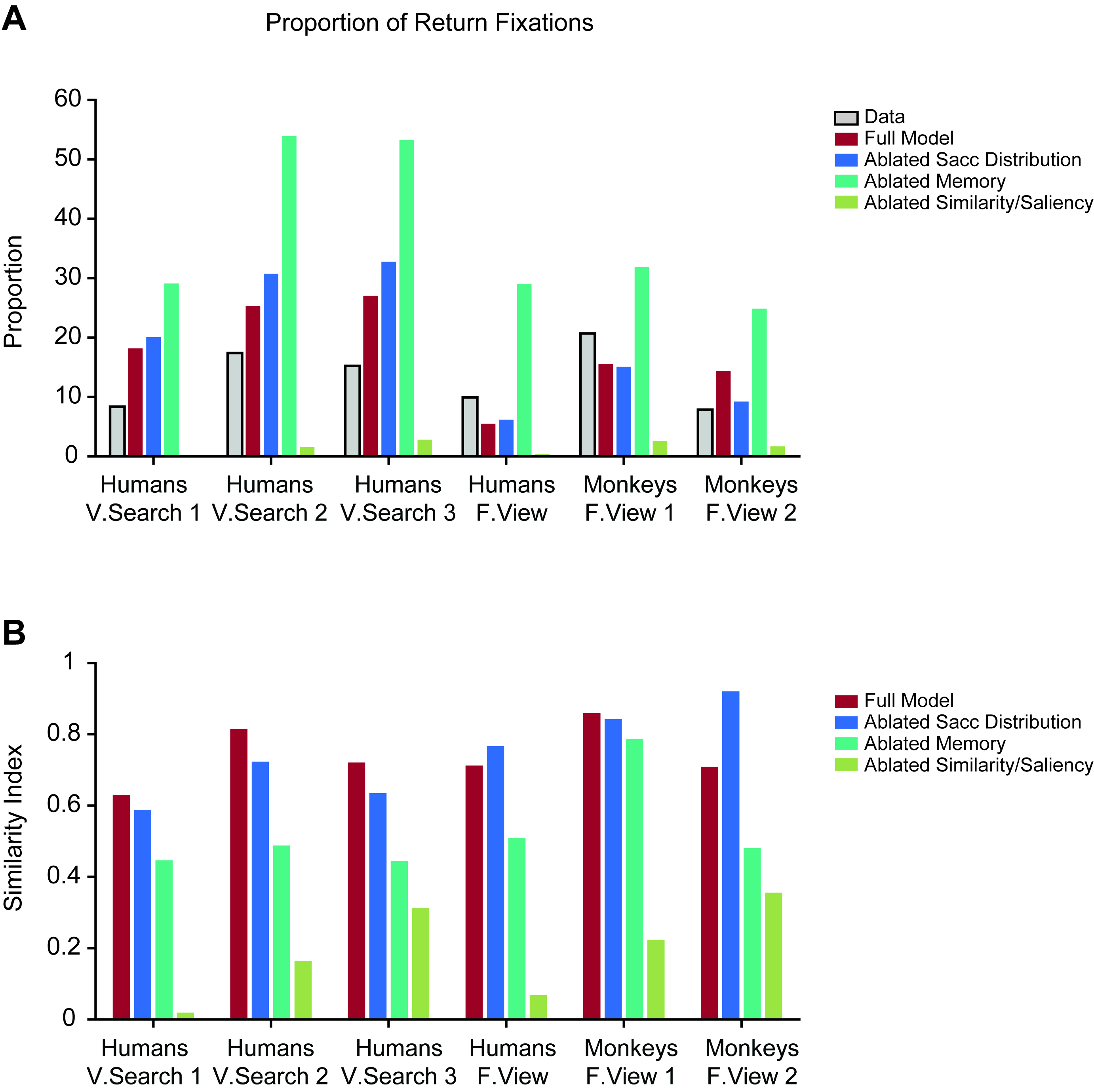

Supplement: S16 Fig — (TIF) [file pcbi.1010654.s016.tif]

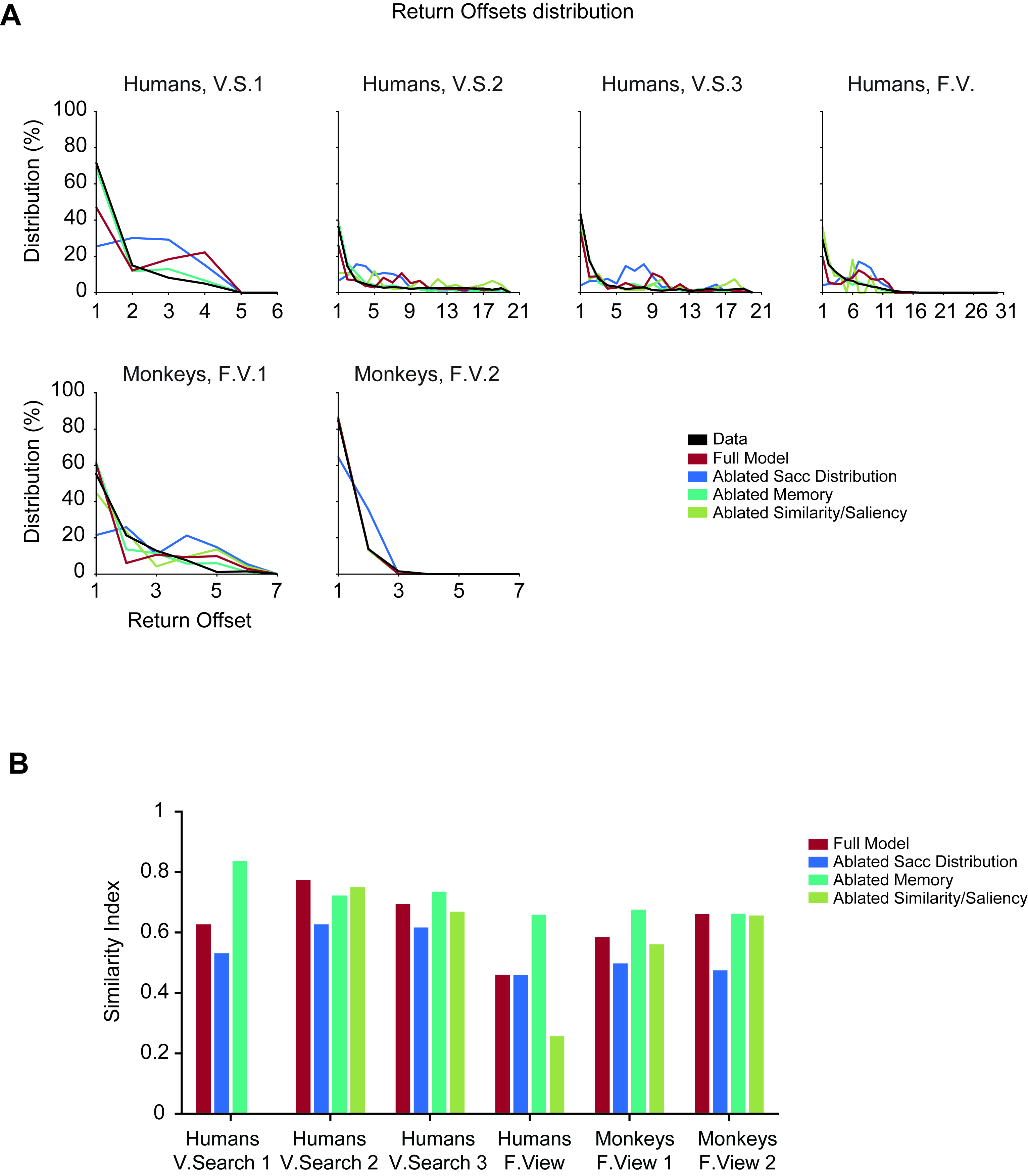

Supplement: S17 Fig — (TIF) [file pcbi.1010654.s017.tif]

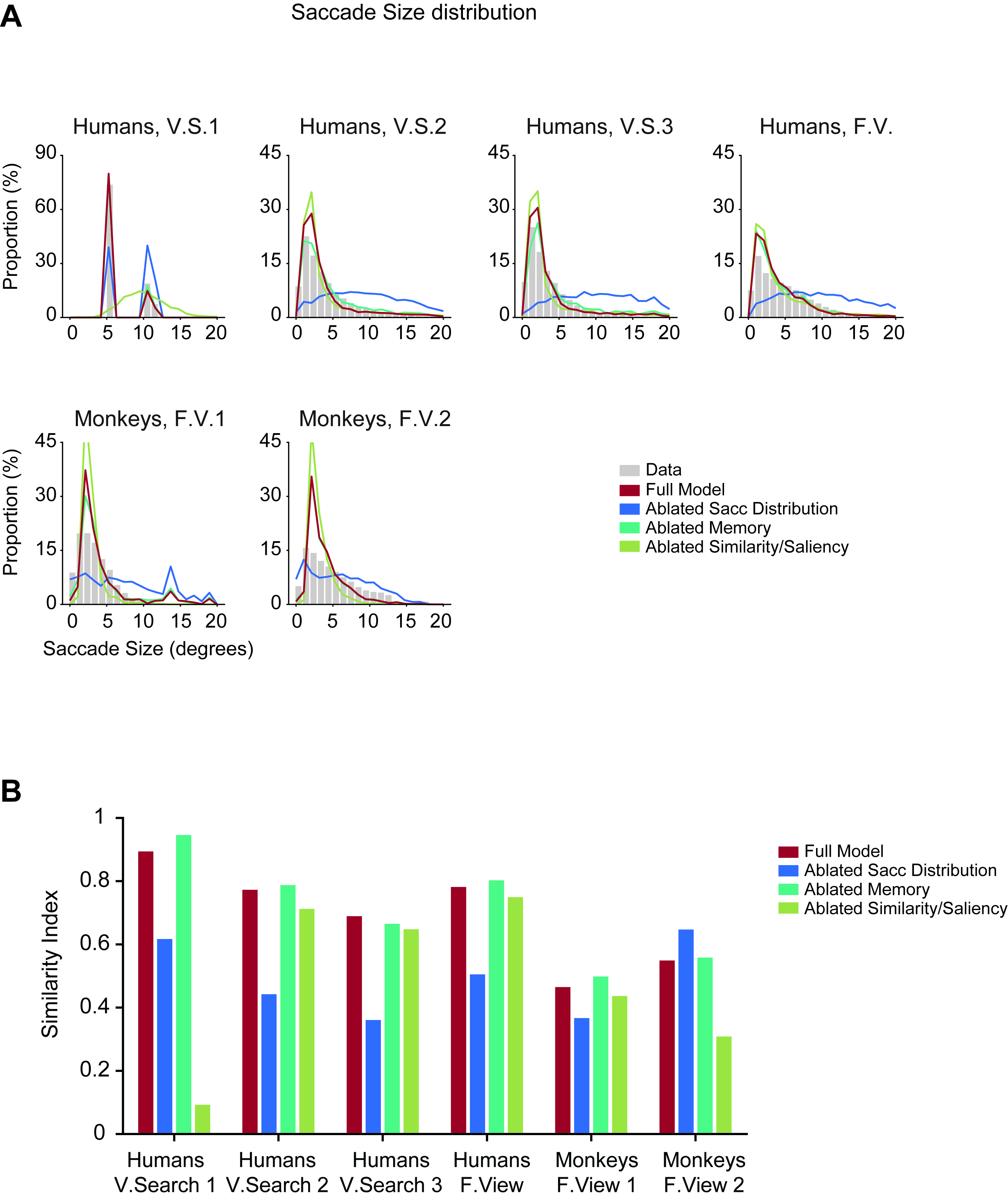

Supplement: S18 Fig — (TIF) [file pcbi.1010654.s018.tif]

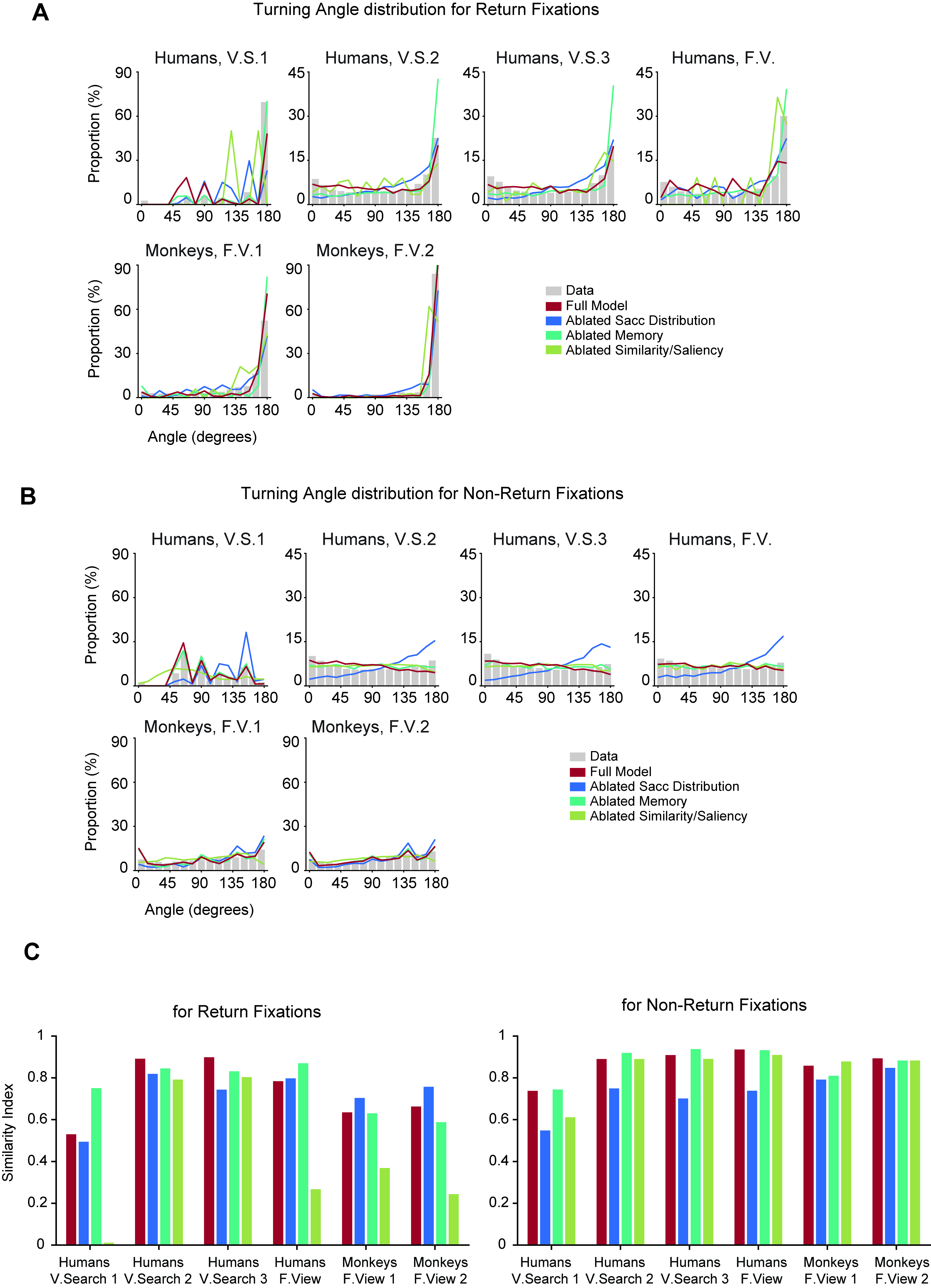

Supplement: S19 Fig — (TIF) [file pcbi.1010654.s019.tif]

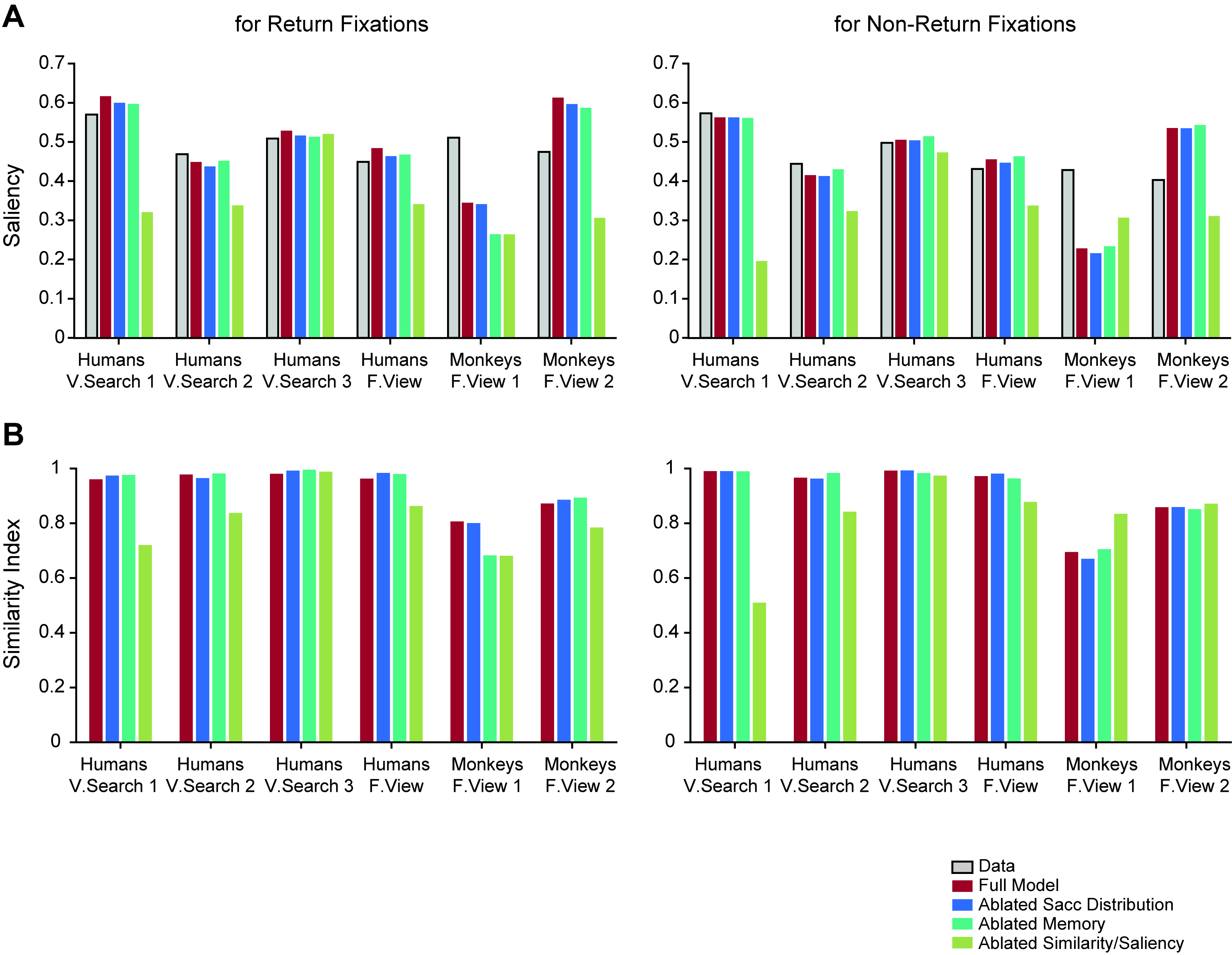

Supplement: S20 Fig — (TIF) [file pcbi.1010654.s020.tif]

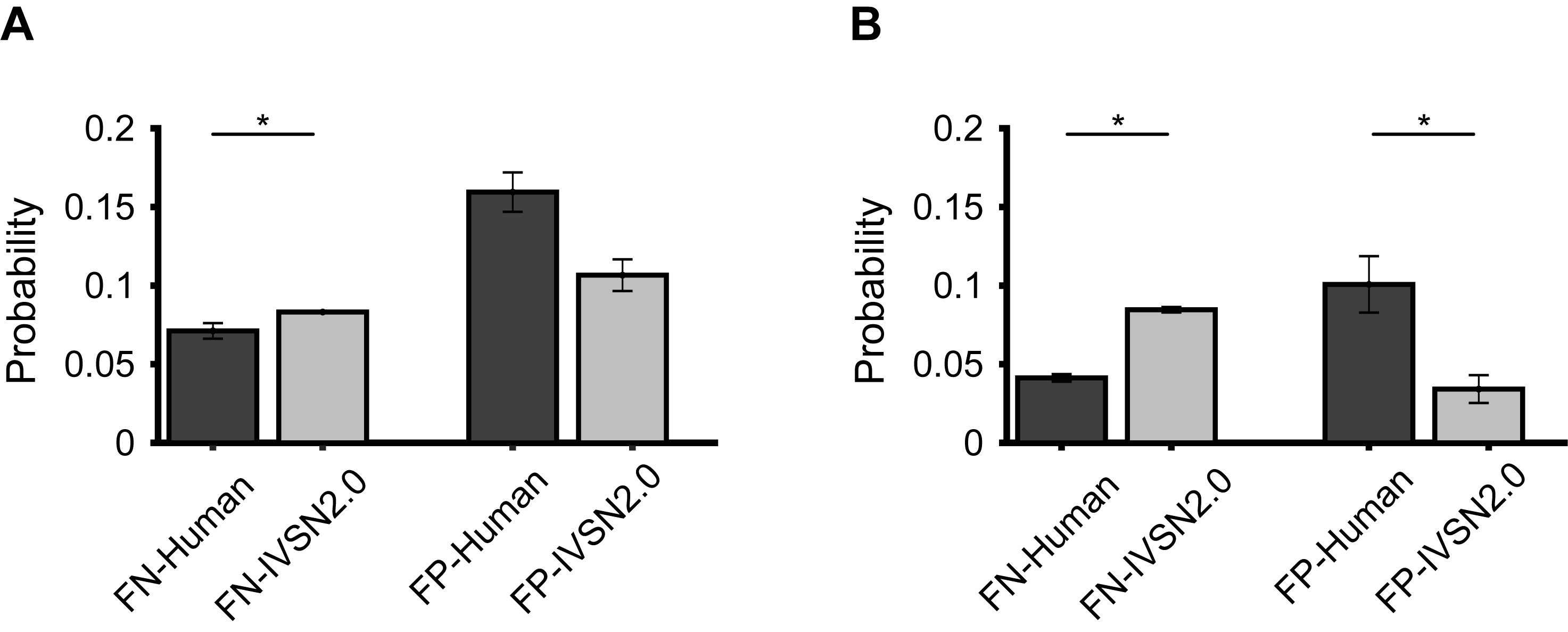

Supplement: S21 Fig — (TIF) [file pcbi.1010654.s021.tif]

**A**

## Proportion of Return Fixations (1 dva)

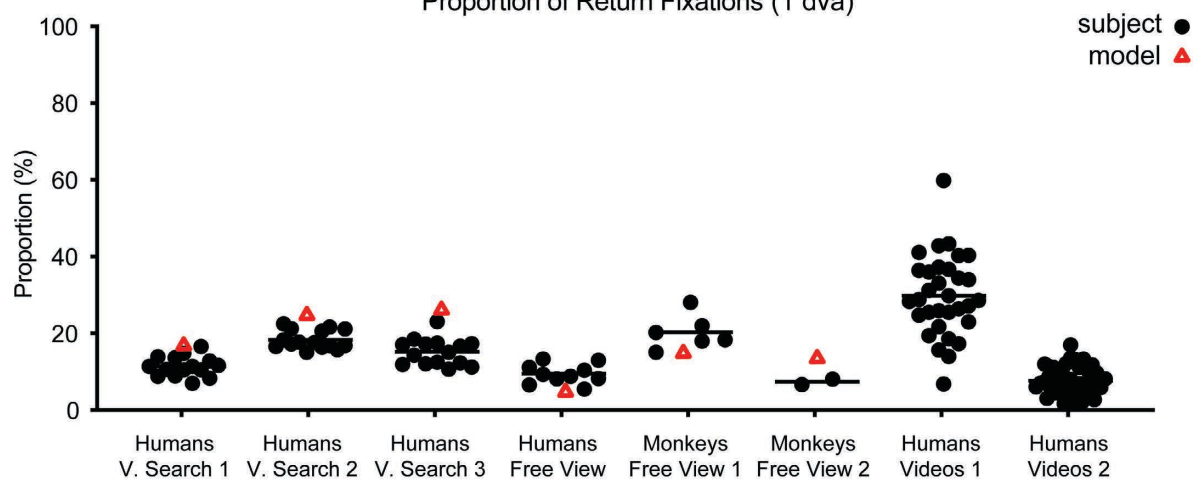**B**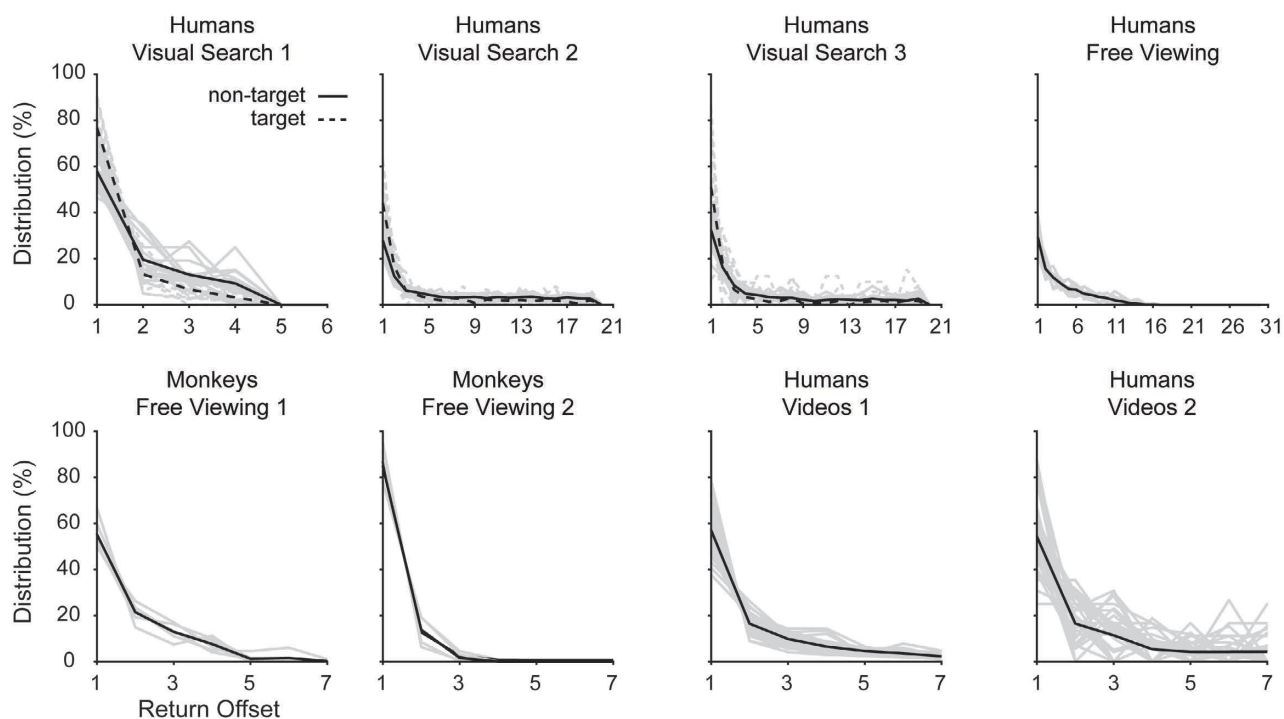**C**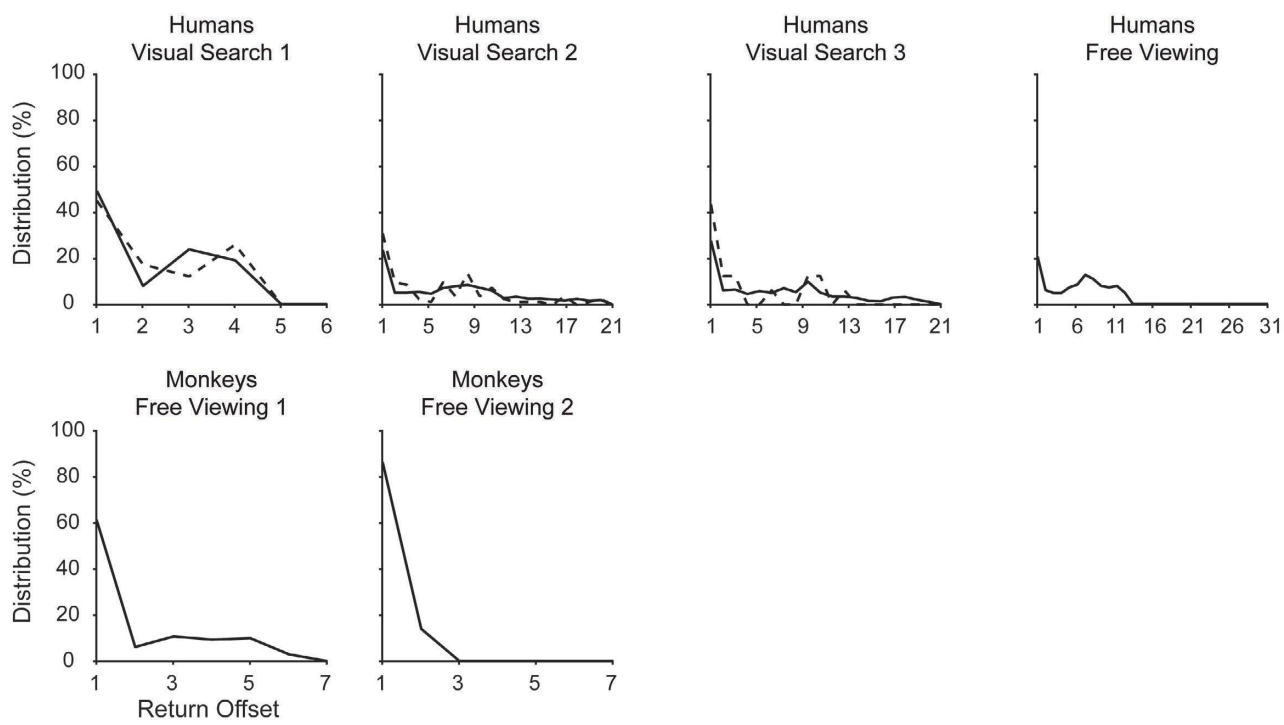

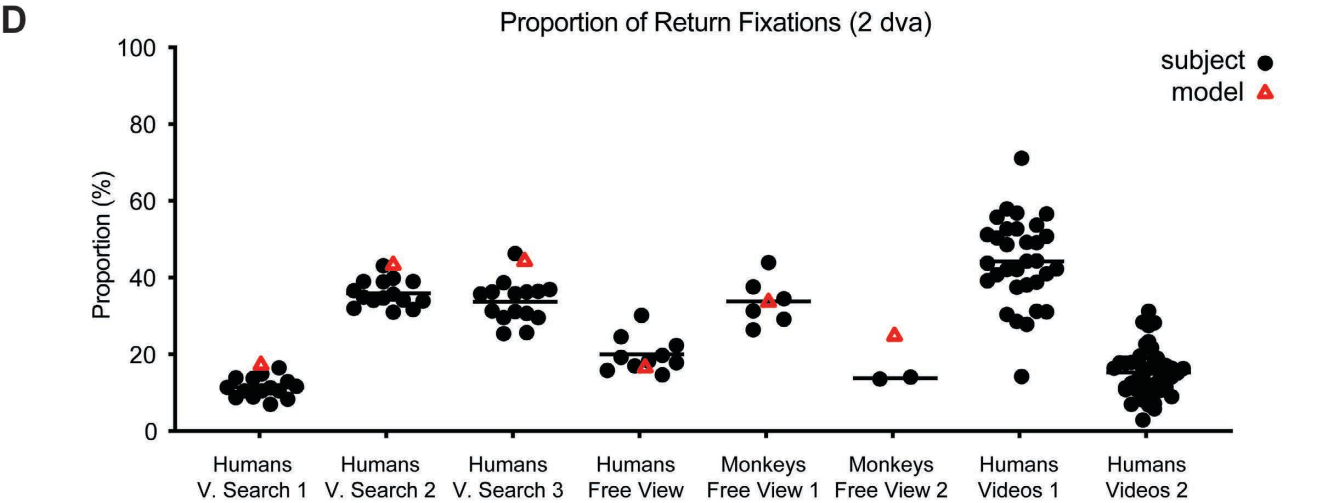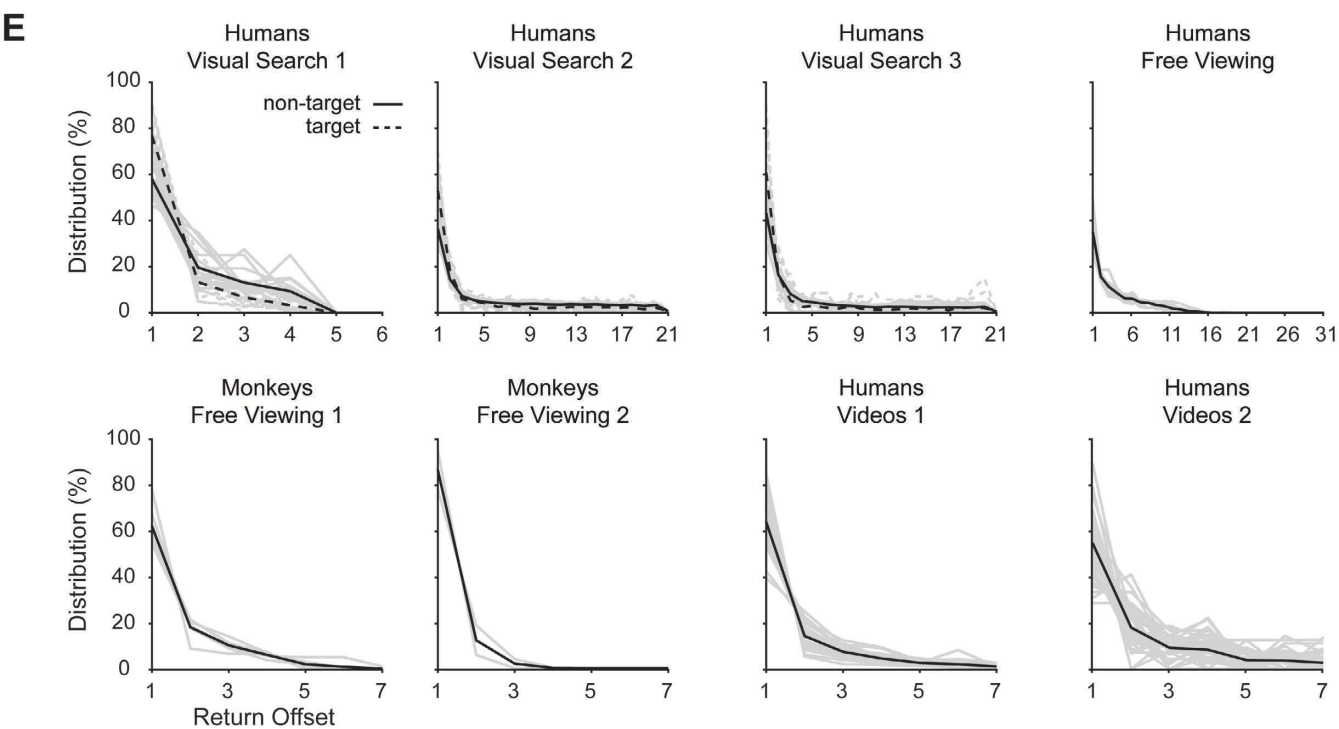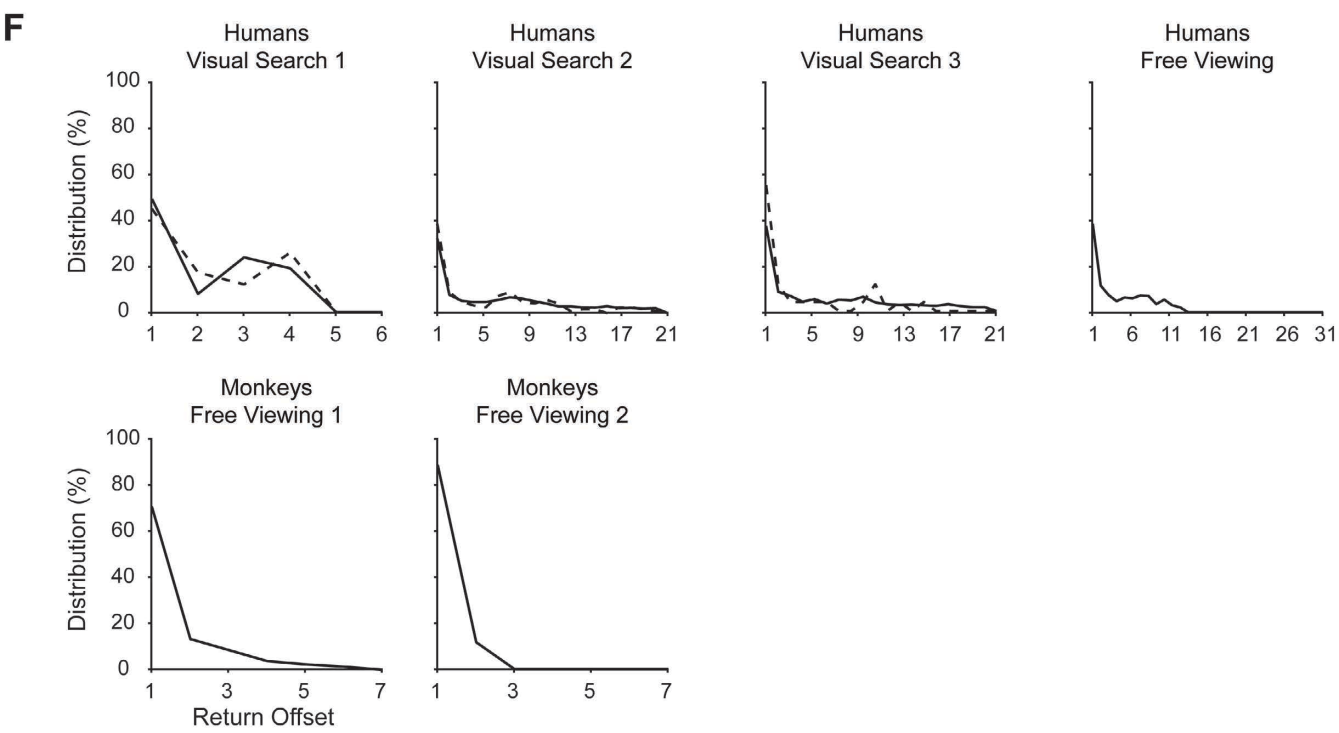

**G**

## Proportion of Return Fixations (3 dva)

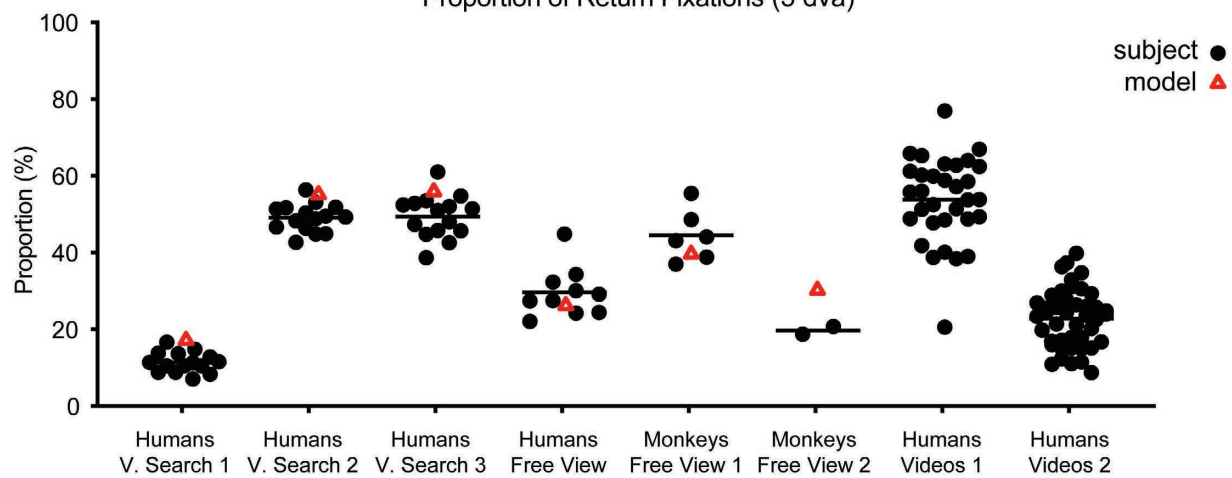**H**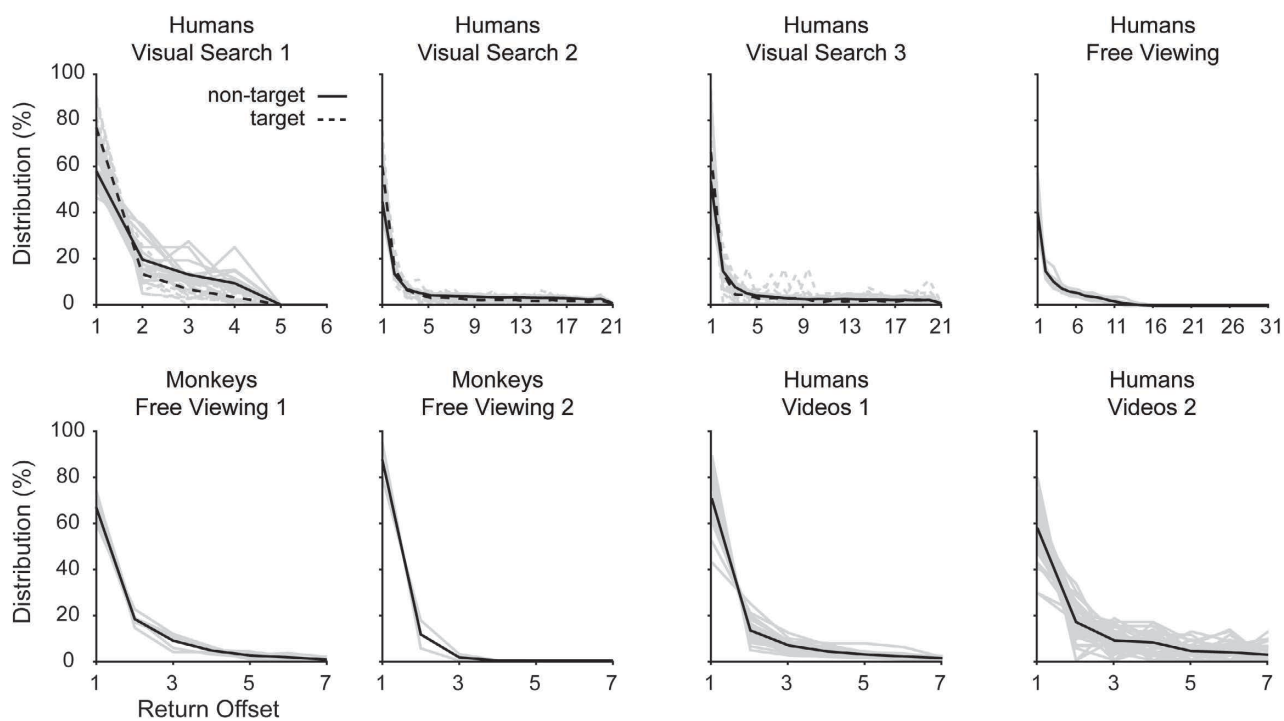**I**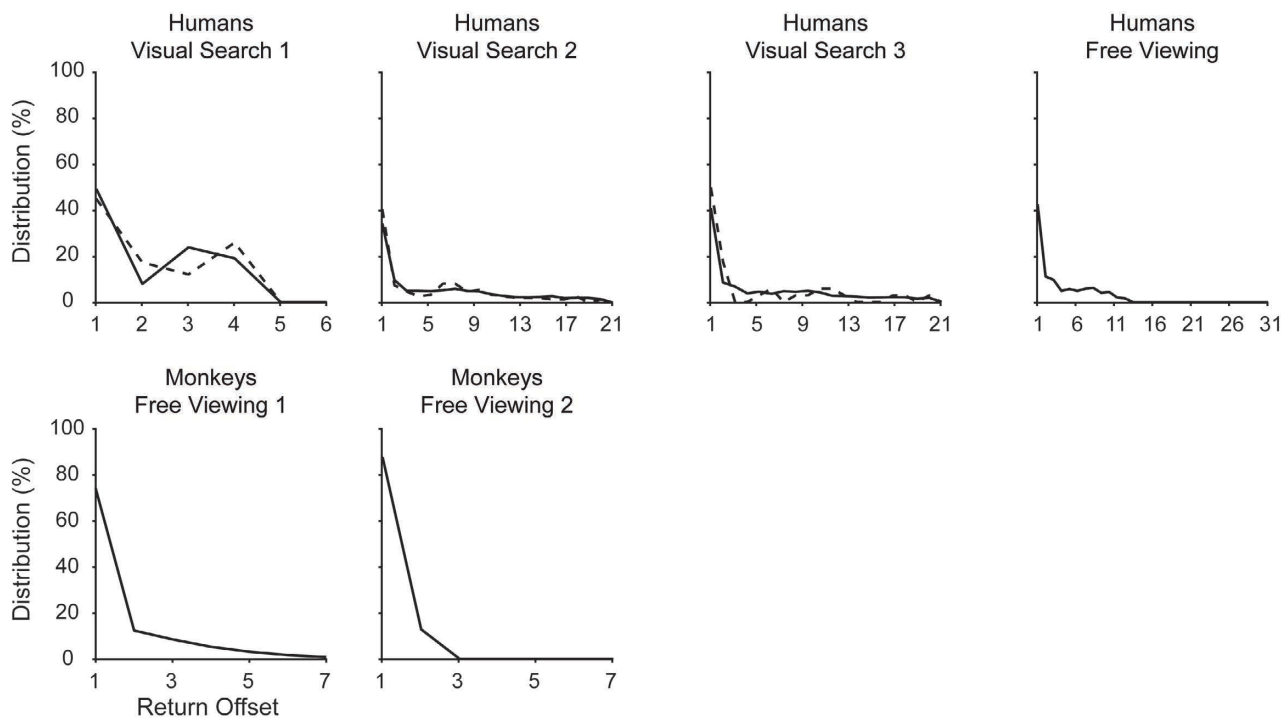

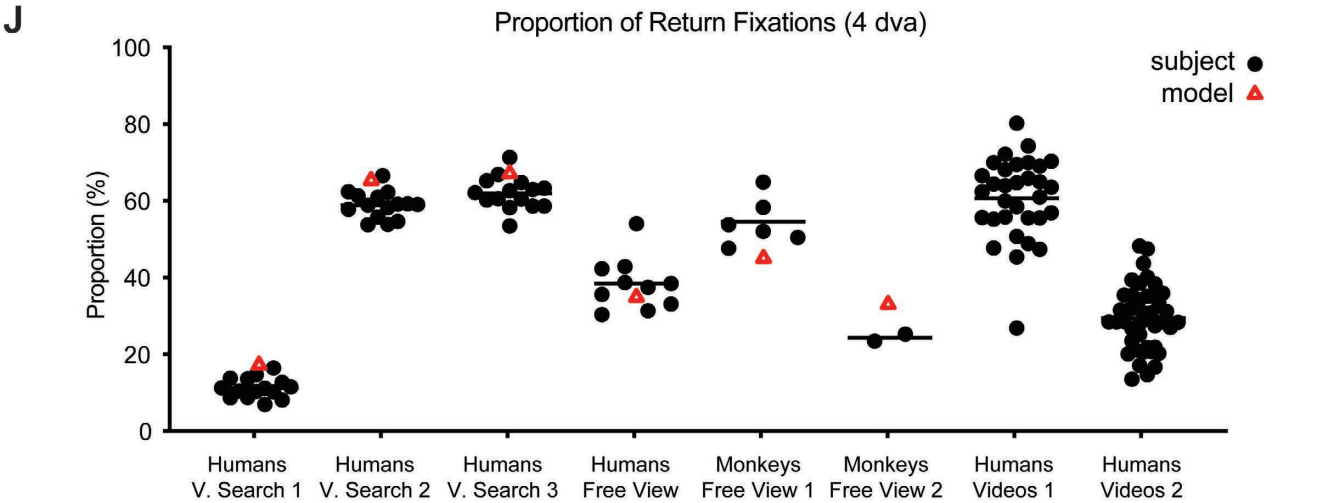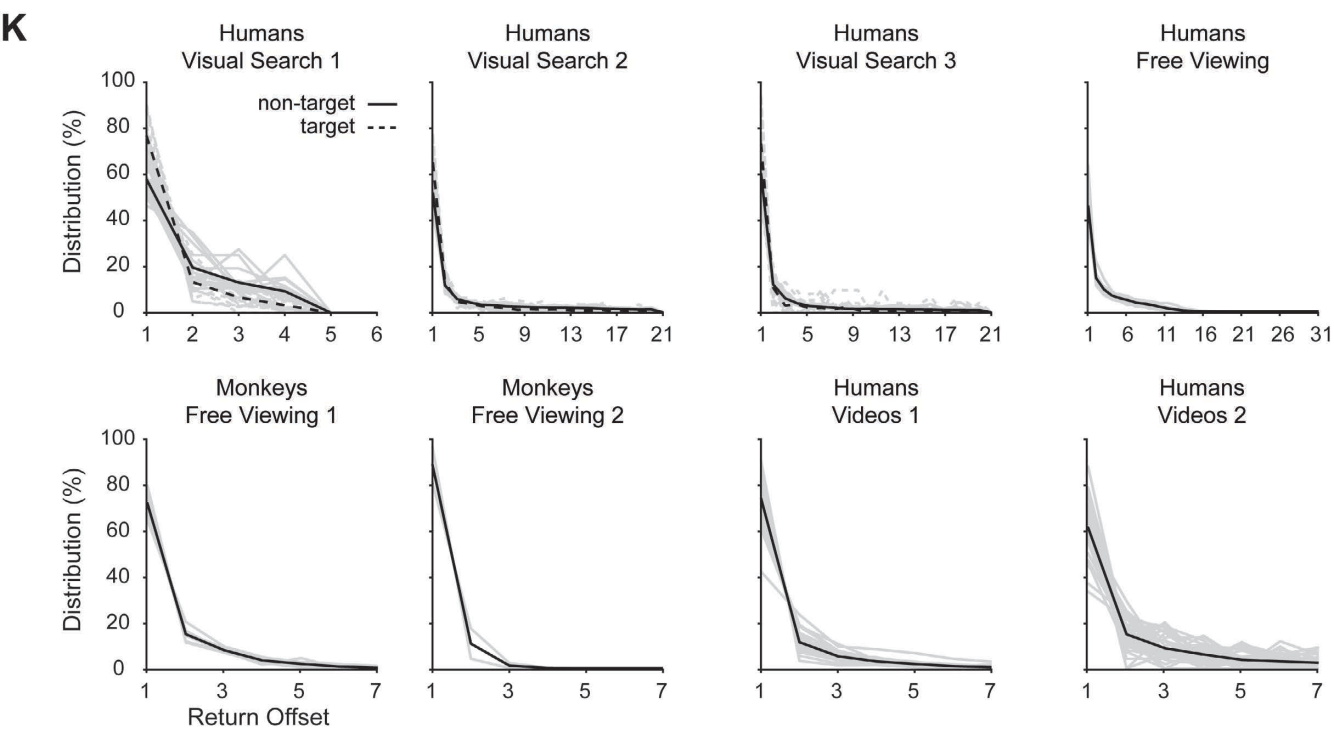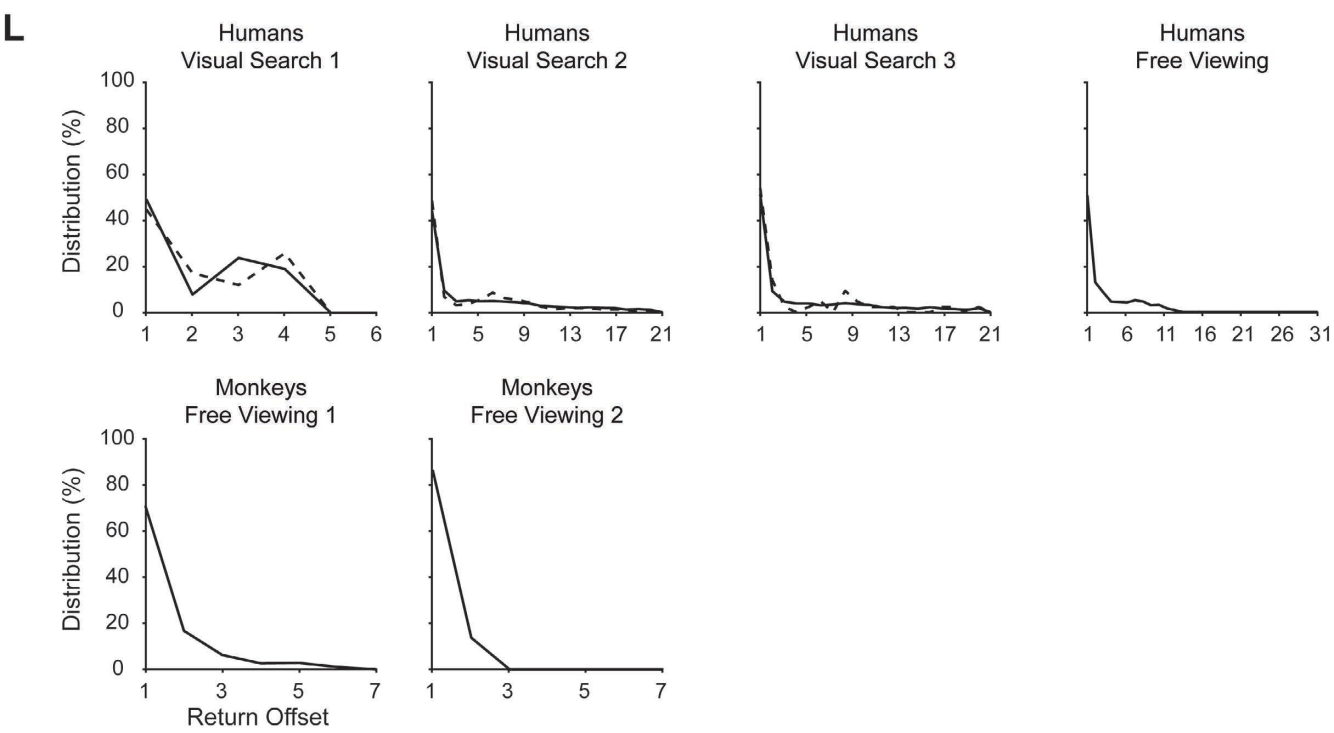

Supplement: S22 Fig — (PDF) [file pcbi.1010654.s022.pdf]

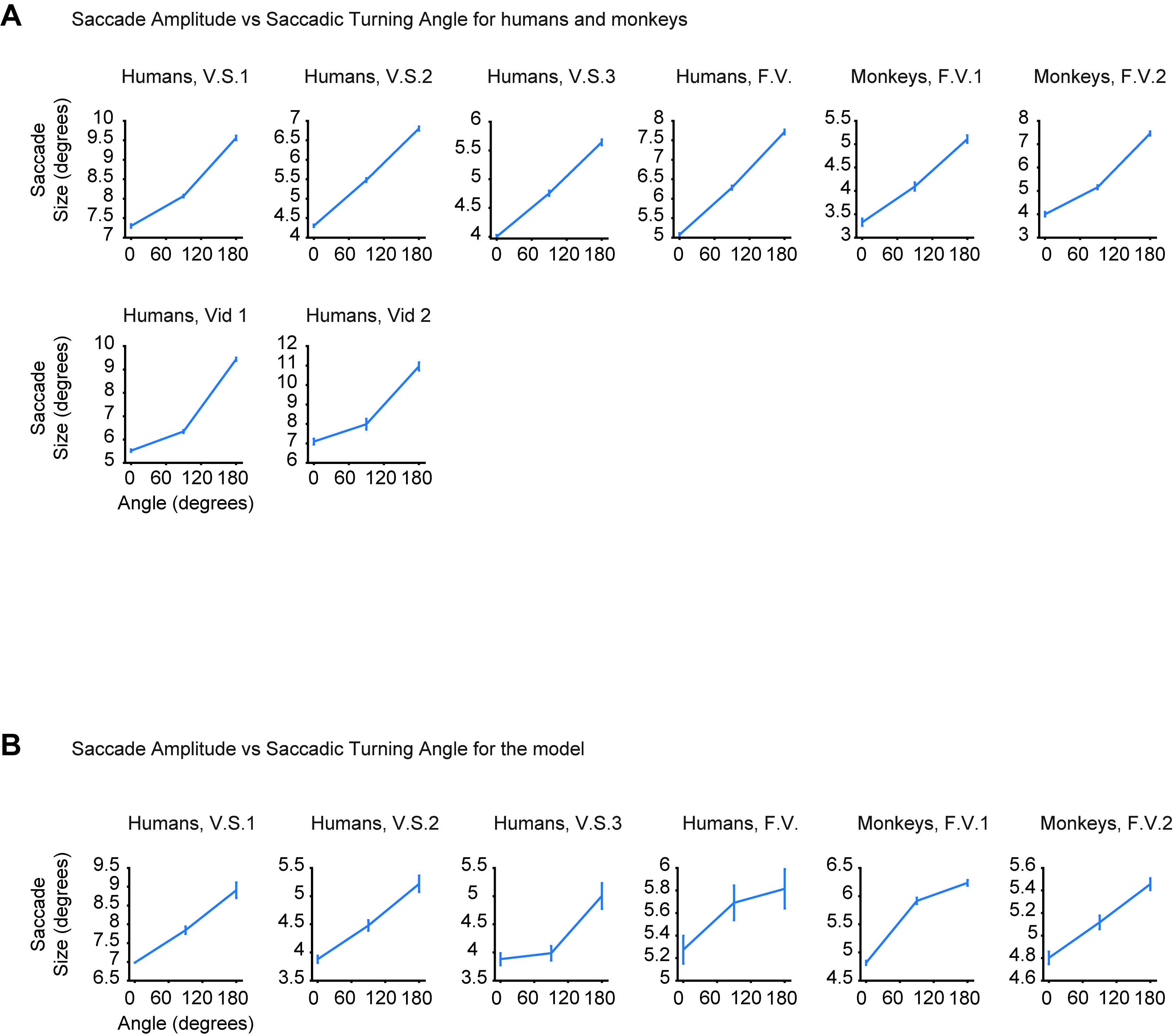

Supplement: S23 Fig — (TIF) [file pcbi.1010654.s023.tif]

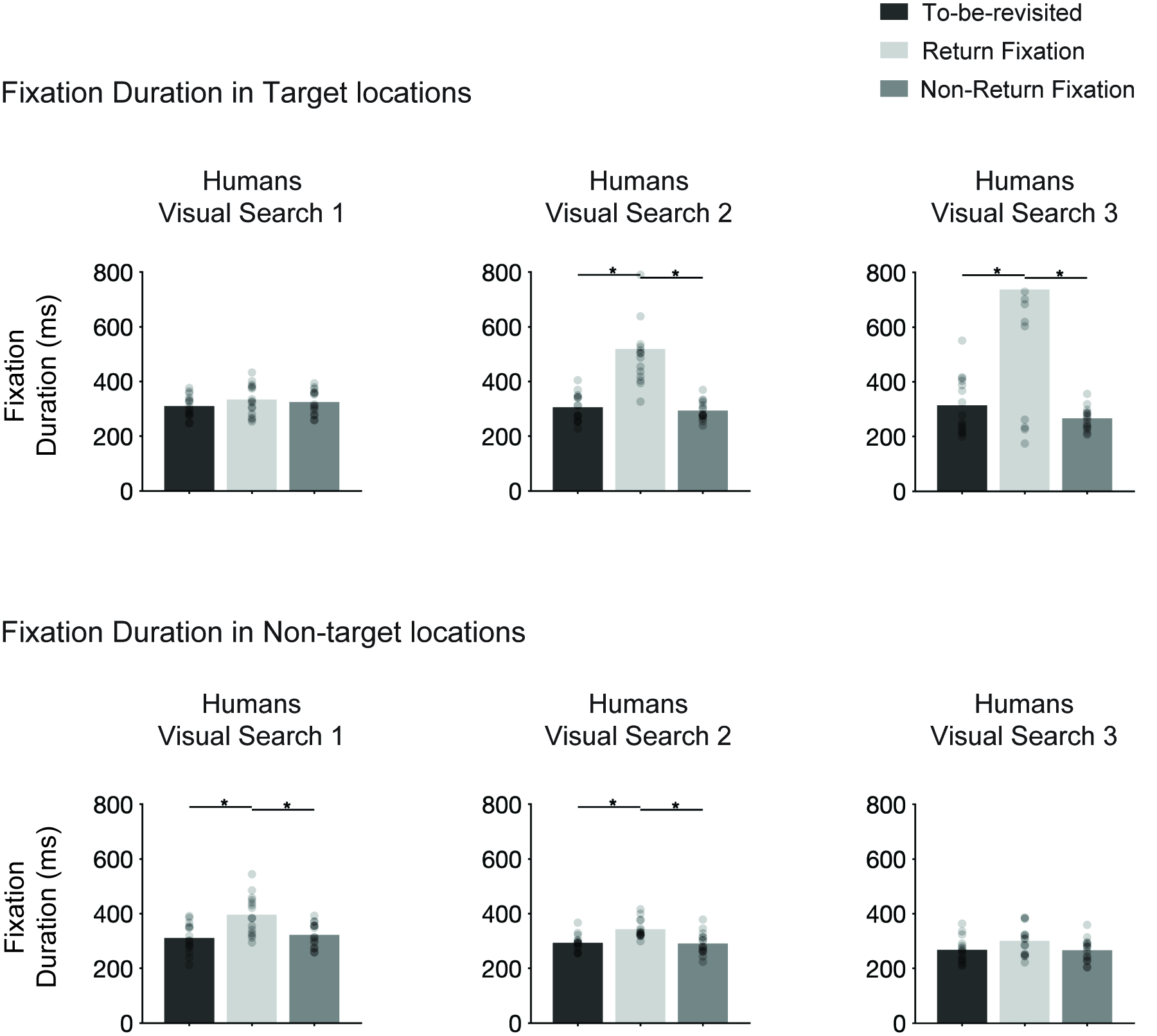

Supplement: S24 Fig — (TIF) [file pcbi.1010654.s024.tif]
