# Supplementary material for: Look twice: A generalist computational model predicts return fixations across tasks and species
Source: PLoS Comput Biol. 2022 Nov 22;18(11):e1010654. doi: 10.1371/journal.pcbi.1010654 (PMC9681066; doi:10.1371/journal.pcbi.1010654)
Supplement: S8 Fig — (PDF) [file pcbi.1010654.s008.pdf]

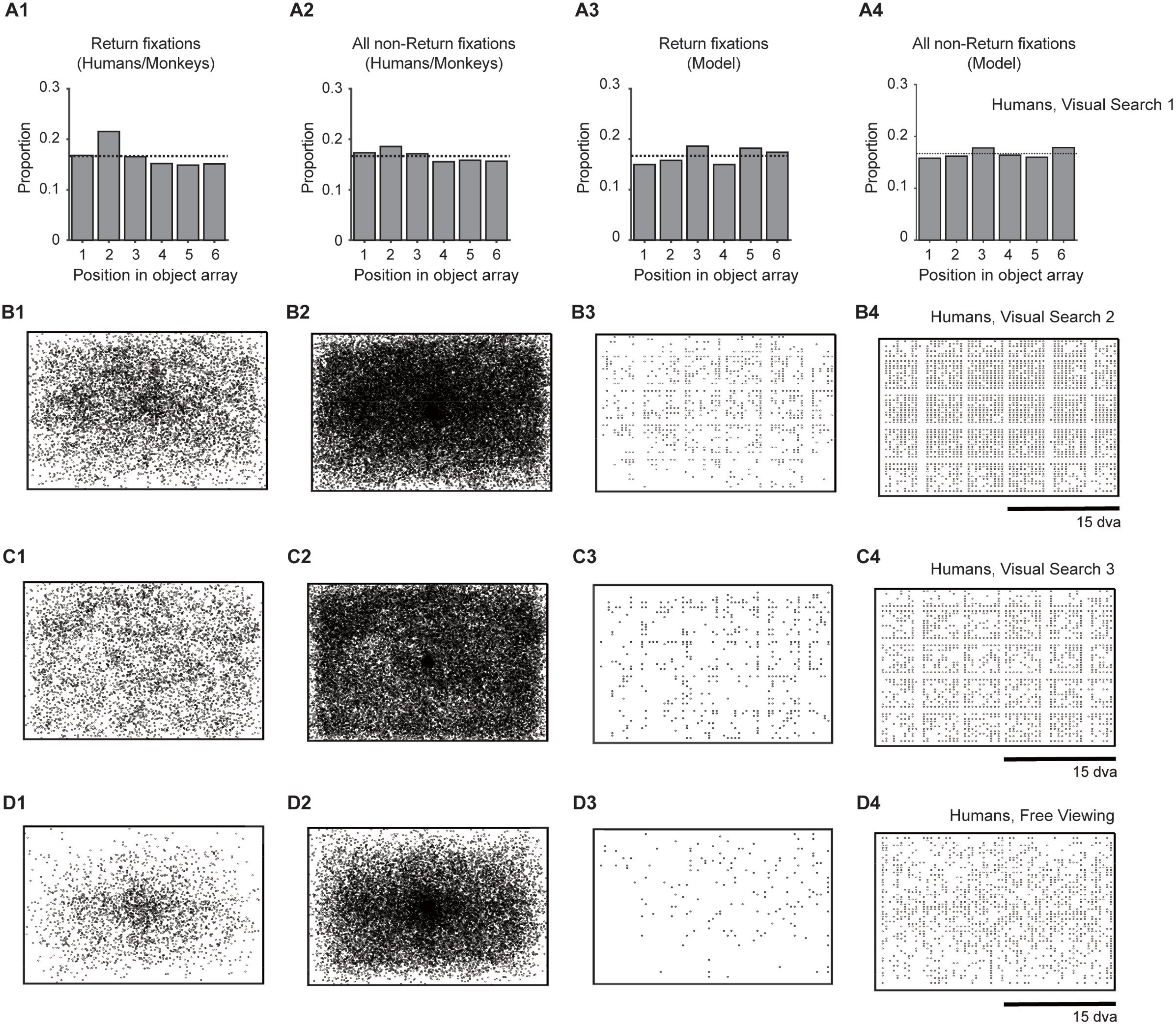
A1
Return fixations (Humans/Monkeys)
A2
All non-Return fixations (Humans/Monkeys)
A3
Return fixations (Model)
A4
All non-Return fixations (Model)
Humans, Visual Search 1
Proportion
Position in object array
B1
B2
B3
B4
Humans, Visual Search 2
15 dva
C1
C2
C3
C4
Humans, Visual Search 3
15 dva
D1
D2
D3
D4
Humans, Free Viewing
15 dva

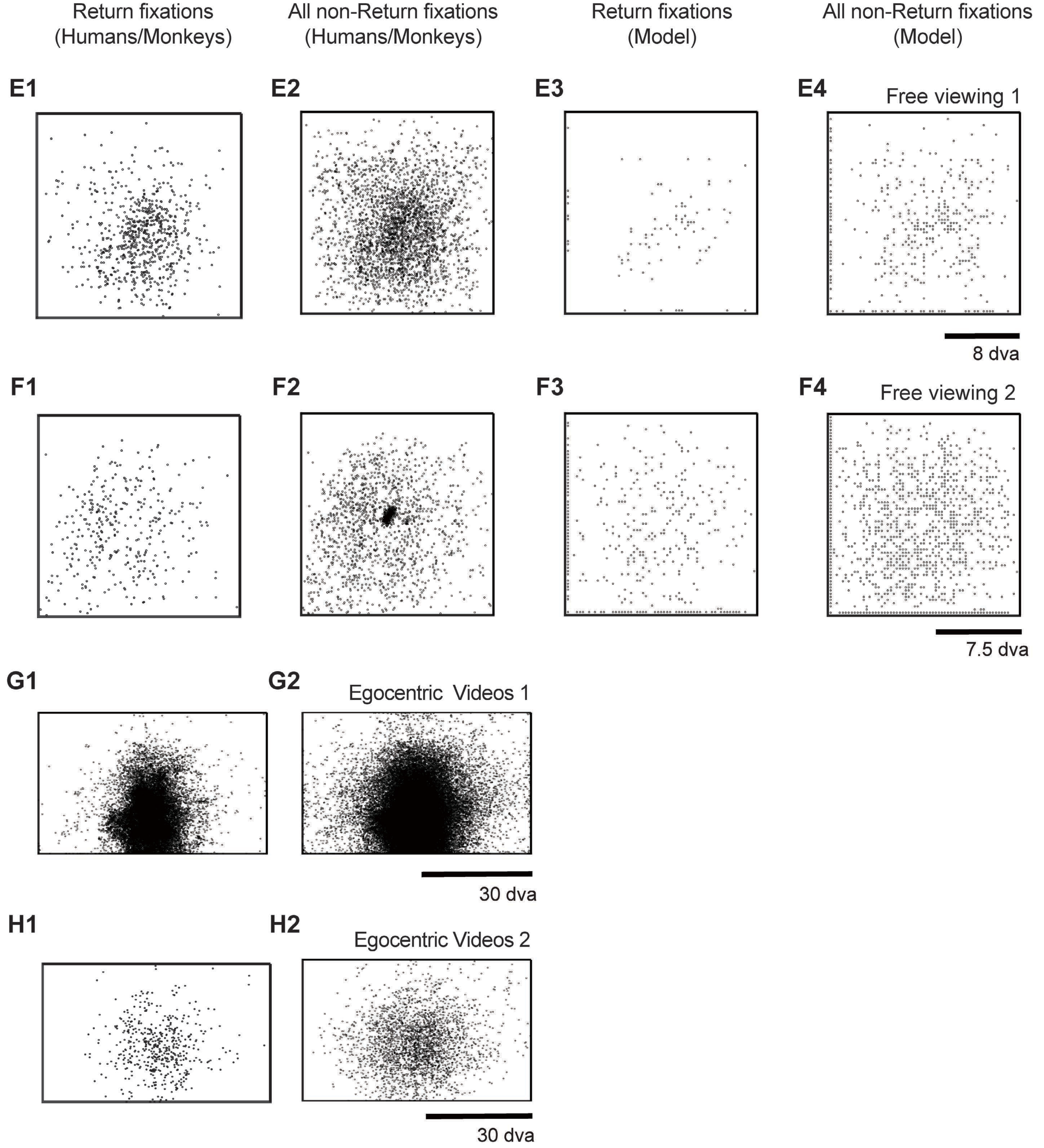
Return fixations (Humans/Monkeys)
All non-Return fixations (Humans/Monkeys)
Return fixations (Model)
All non-Return fixations (Model)
E1
E2
E3
E4
Free viewing 1
8 dva
F1
F2
F3
F4
Free viewing 2
7.5 dva
G1
G2
Egocentric Videos 1
30 dva
H1
H2
Egocentric Videos 2
30 dva
