# Supplementary material for: Look twice: A generalist computational model predicts return fixations across tasks and species
Source: PLoS Comput Biol. 2022 Nov 22;18(11):e1010654. doi: 10.1371/journal.pcbi.1010654 (PMC9681066; doi:10.1371/journal.pcbi.1010654)
Supplement: Attachment — Submitted filename: CompBio_ReviewPointByPoint.pdf [file pcbi.1010654.s025.pdf]

Point By Point Responses

Text in small black font shows the reviewers' questions.

Text in blue indicates our responses.

We are enclosing a version of the manuscript with tracked changes and a "clean" version without tracked changes. Line numbers and figure numbers below refer to the new clean version.

Reviewer #1:

R1.1 In this report, Zhang et al. provide both a detailed empirical description of and computational model of return fixations in humans and monkeys across a variety of visual tasks. The authors show that return fixations are quite common and not unique to specific stimulus configuration or task instructions. They show that a model of visual processing, which incorporates stimulus salience, task relevance of features, along with working memory and constraints on eye movements can produce fixation sequences that also predict that return fixations would also occur. Together, the authors suggest that return fixations can be considered in a framework related to the balance between exploration and exploitation.

In general, I found the paper to be well written and to provide a comprehensive study of the phenomenon of "return fixations". To the extent that the paper's aim is to demonstrate the ubiquity of these I think it succeeds. I do have concerns that in the present form that the modeling component does not add as much to the story as it could. In particular, despite the clear methodological description of the model itself in the latter parts of the manuscript, the impact of the model in terms of providing key insights or conceptual advances is modest. Tied to this concern is the potentially interesting connection to "explore and exploit". But here again, I expected the model to make particular predictions about this balance. Instead, from my reading, the model is essentially used to show that a combination of constraints (admittedly including some elements not present in basic saliency models) would predict that return fixations should occur. But again there seem to be no real key insights about what the model predicts about behavior more generally. I think the paper falls somewhere between an empirical paper and a computational paper, but doesn't effectively merge these as well as it potentially could.

We thank the reviewer for these important comments. We have now removed the discussion of exploration and exploitation, as suggested. We have accordingly modified the abstract, introduction, and discussion. The goal is to demonstrate that a simple neural network model consisting of four key constraints (saliency, target similarity, memory, saccade size constraint) plus a generic image feature extractor ("ventral visual cortex", here VGG16 including top-down modulation for target features during visual search), can reveal return fixations and approximate universal and fundamental properties of return fixations. These properties include return fixation frequency, offsets, turning angles, saccade size distribution, and image location preferences. **Critically, the model can approximate these properties across tasks, species, and conditions, without any retraining or parameter fitting**.

Variations of this type of model have been shown to approximate visual search behavior more generally (e.g., Zhang et al Nature Communications 2018, Gupta et al NeurIPS 2021, Adeli et al, CVPR 2018, among many others). Also, other variations have also been shown to approximate eye movements during free viewing (e.g., Kümmerer et al ICLR 2015, ICCV 2017, Schwetlick et al 2020, among many others). These previous efforts do not provide image-computable models of return fixations. We further speculate that return fixations play an important role during object recognition (see also R1.6 below) and we expect that the current model can provide inspiration towards building better models of visual search and object recognition, but this is beyond the scope of the current work. As the reviewer suggested, we included analyses of model ablations to assess the contribution of the different model components to return fixations (**Fig. S16-S20,** [lines 432-461, 485-486. 491-492, 498-499, and 894-914]).

R1.2 From the Introduction, I think it would be helpful to include some "significance" to the last paragraph. The authors write: "We show that both monkeys and humans make frequent return fixations with few intervening saccades, especially but not exclusively during visual search..." And then they say that they want to "gain insights into the mechanisms", they look to their refined model. But what are the key takeaway insights? Are there ways to characterize optimal return fixations proportions based on parameters of the model or manipulation the likelihood of their occurrence? If subjects were guided to slow down their eye movements, e.g., would this reduce the proportion of return fixations observed?

We have emphasized the significance of the findings in the last paragraph of the introduction, as suggested [lines 68-93].

We suspect that the "optimality" of return fixations can only be defined within the context of specific tasks, including instructions, rewards, amount of clutter in the visual field, and task goals. Indeed **Fig. 3A-H** shows that there is quite a large range in the proportion of return fixations. While we expect that there could be multiple additions to the model to incorporate different aspects of each task, we find it rather remarkable that the model can approximate the properties of return fixations across species, datasets, tasks, and conditions, *without any fine-tuning*.

Consistent with this task-dependence, the reviewer also raises an interesting question about slowing down eye movements. Here we studied return fixations under "natural" circumstances including free viewing and free eye movements during visual search. We *speculate* that instructing subjects to slow down their eye movements might lead to changes in return fixations, as the reviewer suggests, but we did not test this. After spending a long time fixating on one location, participants might consider this part of the image sufficiently explored, discouraging a future return to that location (reducing the proportion of return fixations) or delaying return fixations (increasing return offsets). We have added a discussion of the importance of task instructions, including the example suggested here by the reviewer [lines 512-523].

R1.3 As an example of a question that seems appropriate for the model to address, and for which the empirical data might provide guidance, would be the degree to which different parameters could effect the likelihood of return saccades. In particular, it seems like the memory component in the model is one place the authors might consider including the possibility that the prior location doesn't effectively get added into the memory. As opposed to a global decay, is it not plausible that specific locations fail to get added (perhaps as a function of something else, like stimulus complexity or fixation duration). In any case, while I can't say I have specific hypotheses about the relative contributions of the target similarity factor or the memory decay, it would make the inclusion of the model stronger if it did more than predict that inclusion of the extra terms led to return saccades.

This is also a great suggestion. To address this question, we introduced new modified versions of the model in which the different components were ablated. We characterized the relevance of each of the four key modules in the model by generating new fixation sequences using ablated models and comparing their performance to the default model and to the experimental data (new **Figs. S16-S20**). We also added a new section to the text to discuss these new analyses [lines 432-461]. Briefly, altering the contribution of the memory and saliency/similarity components distorted the proportion of return fixations generated by the model. The first case considered the possibility that prior location does not effectively get added into the memory. A defective memory module caused the proportion of return fixations to be too high, producing frequent return fixations after short offsets. In the second case, the absence of relevant locations in the image (provided by saliency/similarity maps) resulted in few return fixations. We describe these results in [lines 432-461] and **Fig. S16-S20**; and the methods to implement the model variations in [lines 894-914].

R1.4 Regarding the model fit, the assessment of the quality of the model fit seems a bit arbitrary. For example, in Figure S10 the caption reads "The distribution of saccade sizes in the model matches the one from humans and monkeys", but the first in 2-6 don't seem good at all (and the distribution in 1 is highly unusual?). In any case, the loose use of the term "matches" would seem to undermine the inclusion of the model as anything more than a vague proof of concept.

With respect to former **Fig. S10A** (now **Fig. S6A**, column 1), the Human Visual Search 1 experiment involved 6 objects at equidistant positions along a circle (see **Fig. 2A**, and Zhang et al., 2018 for details). By and large, subjects fixated on the objects (as opposed to the gray screen surrounding the objects). Thus, there are only a few discrete possible saccade sizes that dominate the distribution and that is why the distribution appears so "unusual".

The reviewer raises an important point about the use of the word “match” when referring to the comparison between the model and human behavior. It is important to note that we are *not* fine-tuning any parameters in the model to fit the data. There are many parameters that could be tuned, including the weights in the “visual cortex” module and the specific layers selected both of which are critical for the calculation of saliency (there is extensive work on models of bottom-up saliency; e.g., Itti and Koch 2000), which layers are used to provide top-down modulation in search tasks (see Zhang et al 2018, Gupta et al 2021 for discussion of this point), the memory decay function (**Fig. S10A**), the saccade size prior maps (**Fig. S10B**), the threshold to define return fixations (**Fig. S22**), and the relative contributions of the different modules. Fine-tuning model parameters in each task yields better quantitative fits to the data. Instead, we consider the model to represent a zero-shot, proof-of-concept, demonstrating that the four key ingredients of the model (plus the image feature extraction) can *qualitatively* account for the fundamental properties of return fixations across species, tasks, dynamics, with zero free parameters, without any retraining or fine-tuning [lines 538-555]. We explicitly mention that the model does not always quantitatively match the experimental data [lines 546-550, 579-580].

R1.5 I think the comparison of return fixations across task is a positive, but I'm a little confused about how to think about return fixations in a dynamic video? As an extreme case, would we be surprised to show that drivers make return fixations when driving (as they repeated look at the controls on the dash?). The cooking video would seem to fall into this camp.
We agree with the reviewer. We suspect that subjects make frequent return fixations during cooking, for example, to ensure that they do not cut their fingers while slicing a carrot or to grab the right appliances. We are not aware of other studies showing return fixations in this type of first-person video, and we think that it is interesting to document this phenomenon. However, we are not trying to convince the reader that this should be surprising. We *suspect* that we make many return fixations during natural viewing behavior in the real world.

R1.6 There is also a missed opportunity to consider the dynamics of this process, which seems so critical to this problem (this is noted by the authors on page 9). In the Introduction, the suggestion is made that "return fixations could constitute a useful strategy during visual search, especially in difficult tasks" (p. 3) but it's not clear that "useful strategy" is the best term here? It seems like the issue is more of a mismatch between fixation duration and the inability to extract relevant information in time, so the system is required to overcome these misses? It may be a semantic thing, but does seem to highlight the possibility of exploring how the proportion of return saccades depends on saccade rate.
We agree with the reviewer and we are especially interested in this point. We removed the term “useful strategy”, as suggested. We now include the points made by the reviewer, almost verbatim (thank you!), in the Discussion [lines 524-537]. Unfortunately, by and large, we can only speculate about these issues. The indirect evidence that we have in support of this argument is the case of visual search. Consider the case of looking for Waldo. Subjects can fixate on Waldo, but often they continue the visual search process and move their eyes away, and only come back later to Waldo and click on it, indicating that they have found it (Zhang et al 2018). We argue that it is highly unlikely that they consciously recognize Waldo during the first fixation but decide to keep moving their eyes, look at other places, and then come back. This observation is not restricted to Waldo searches but also happens in natural images and even in searches within object arrays (Zhang et al. Nature Communications 2018, see also Sheinberg and Logothetis J. Neuroscience 2001 for a similar point in monkeys). While this is an indirect argument, we think that this suggests that conscious recognition fails during the first fixation (“inability to extract relevant information” in the way expressed here by the reviewer).
As the reviewer suggests also in R1.2, manipulations that impact the saccade rate could alter this process and thus change the proportion and properties of return fixations.

Specific comments

R1.7 Analysis: It's not clear what the authors are actually comparing in the reported statistical tests (I do see the short description on page 21). For example, on page 3: "the proportion of return fixations was higher than expected by a null model implementing random eye movements while respecting the distribution of saccade sizes (p < 10-15, two-tailed t-test, t < -16, df = 1, 598, Methods)". Where are these degrees of freedom coming from and is a t-test really appropriate here? For these analyses it seems like all fixations are just collated into a single large analysis. I would have thought a more appropriate analysis would have been to use each subject's average as a datapoint? To that end, the bar charts in Fig 3 (and S1, S2, and S4) could be composed on single points from each subject, which would provide a more robust analysis of these data, demonstrating that all subjects make return saccades (which I suspect is the case).

We have redone all the statistical analyses, as suggested by the reviewer. We describe the new statistical analysis methods in [lines 1099-1106]. We now report statistical tests *across subjects*, instead of *across fixations*. As suggested by the reviewer, we considered each subject's average as a single data point and performed statistical tests to assess the statistical significance of the proportion of return fixations. Triggered by this comment, we also ran new statistical tests across subjects for saccade sizes, fixation durations, and saliency. We created new figures to reflect the new analyses, where we show data for each individual subject (**Fig. 3, 4, 6, S1, S2**).

We would like to clarify two additional points. First, the entropy measure used to assess *between-subject* consistency is, by definition, a calculation across images combining subjects (**Fig. S4**). Hence, we kept the previous analyses for **Fig. S4**. Second, the rationale for the previous analyses was based on the work in monkeys, where we have very few individuals. The change in the statistical analyses implies that we cannot report accurate statistics across individuals for the experiment where we have two monkeys (e.g., **Fig. 3F**). We could have kept the previous method only for **Fig. 3F**, which showed a significantly higher proportion of return fixations than expected by chance, but we thought that it would be too confusing to mix different statistical methodologies. Therefore, we opted to skip the statistical comparison in **Fig. 3F**.

R1.8 Methods: why is the eye tracker introduced in the "Human free viewing" section and not the "Visual search on static images" above?

We moved the eye tracker introduction to the previous section [lines 621-630].

R1.9 Methods: The choice of 1dva for defining a return saccade seems reasonable, but as this entire paper depends on this parameter, it would have been nice to see that the entire analysis pipeline would lead to the same conclusions with different choices for this value.

We repeated all the analyses using different thresholds to define return fixations (ranging from 1 to 4 dva). We generated new figures for the proportion of return fixations and offsets (**Fig. S22**). As expected, a larger radius threshold leads to more return fixations and shorter offsets. The correspondence between the model and the experimental data was actually slightly better when we considered larger thresholds.

However, we argue that, on average during natural viewing, fixations that are 4 dva apart are not exactly quite focusing on the same "thing". Given that the error in the eye tracker measurements is slightly below 1 dva, it seems that this is a reasonable criterion to define return fixations [lines 1014-1024]. Thus, we kept 1 dva as the criterion to define return fixations throughout the text (except for **Fig. S22**).

R.10 p. 8 "oculomotor" is misspelled "occulomotor"

Thank you. Corrected.

Reviewer #2:

Text in small black font shows the reviewers' questions.

Text in blue indicates our responses.
We are enclosing a version of the manuscript with tracked changes and a "clean" version without tracked changes. Line numbers and figure numbers below refer to the new clean version.

R2.1 The manuscript describes experimental and modeling studies on return fixations during eight image (or movie) exploration/search tasks. The choice between fixating new locations and returning to an old fixation location is conceptualized as a trade-off between exploration of new information and exploitation of previously encountered, but potentially uncertain information. While this trade-off makes intutitve sense, the manuscript does not present compelling evidence for it. Instead, fixation locations in the different tasks are compared to a null model of random fixations. It is found that fixations are not random, which is unsurprising. Furthermore, even when there is no explicit incentive to return to a previously fixated location in the free-viewing tasks, return fixations are still abundant (page 3, 'return fixations are ubiquitous'), which seems to contradict the exploration/exploitation trade-off explanation. Further results are the consistency of the return fixations across subjects, the more frequent returns to salient locations, and locations similar to the target in the search tasks.
These observations inspire an image-computational model, which is the most interesting and novel aspect of the manuscript in my opinion. Feature maps are computed with a pre-trained 'ventral stream' model (VGG-16). These feature maps are used to compute a saliency map and a similarity map (in case of the search tasks). Furthermore, to incorporate IOR a memory map is added to the model, which makes the return to a very recently added location unlikely. Additionally, a 'saccade size constrain' map ensures that the generated saccades match observations without having to implement a complete oculomotor system. While there are plenty of attention models based on saliency and task-relevant information, the addition of a memory map and saccadic size constraints constitutes a novelty, whose importance to the field could be appreciated even more if it was more rigorously evaluated.
Major comments:
- The idea of viewing novel vs. return fixations as 'exploration vs. exploitation' is interesting, but I was not able to find compelling evidence for this distinction in the experiments or the model. On the experimental side, I would have expected reward manipulations to demonstrate that return fixations are indeed exploitative. On the modeling side, a reinforcement learning component to the model might have helped in substantiating this claim. I would like to ask the authors to add data/models to this end, or reconsider the metaphor.
We have taken this comment to heart and we have removed the discussion of exploration versus exploitation. As the reviewer suggested, we focus on the universality of return fixations across tasks, species, dynamics, and on the proposed computational model as a proof-of-concept account of the fundamental properties of return fixations [lines 14-32].

R2.2 - The model deserves more quantitative evaluation before it can be considered for publication. As the authors themselves point out, more relevant null models than a random fixation model could be easily constructed from the computational model, e.g. by removing the memory map and/or the target similarity map. Quantitative model comparison scores for relevant aspects of the paper (Hit rates, return offsets etc.) between humans/monkey and model should then be computed, to demonstrate the benefit of the proposed model architecture over standard saliency models.
The model uses a "ventral visual cortex" image feature extractor plus four key modules to generate fixations: saliency maps, target similarity maps, memory decay, and saccade size constraints. As suggested by the reviewer, we introduced new versions of the model in which the different components were systematically removed. To characterize the relevance of each module in capturing basic properties of return fixations in humans and monkeys, we generated fixation sequences and repeated all the analyses using each ablated model. These ablated models are discussed in lines [894-914] and the results are shown in new **Fig. S16-S20**. We also introduced a similarity index in lines [1092-1100], and **Fig. S16B-S20B** to directly compare the experimental data versus the default model and each ablated model.
First, we found that subtracting the contribution of the memory and saliency/similarity components deteriorated the proportion of return fixations generated by the model. A defective memory module caused the proportion of return fixations to be too high, producing frequent return fixations after short offsets (**Fig. S16-S17**). Second, the absence of relevant locations in the image (provided by saliency/similarity maps) resulted in too few return fixations (**Fig. S16**). Third, we observed that the distribution of return offsets tended to concentrate at higher offset values (between 2-9) when the saccade-size-constraint was

ablated compared to the full model (peaking at 1), which resulted in poor correspondence with the human/monkey data (**Fig. S17**). Fourth, removing the constraint for saccade size led to mainly uniform distributions of saccade size (**Fig. S18**) which do not match the experimental data. Fifth, the worst predictors of turning angle distributions were models from which saccade size and similarity/saliency maps were removed (**Fig. S19**). Finally, the correspondence between the model and behavioral data for saliency was most affected when the similarity or the saliency components were ablated (**Fig. S20**). A new section was added to the manuscript to describe these analyses [lines 432-461]

Reviewer #3

Text in small black font shows the reviewers' questions.

Text in blue indicates our responses.

We are enclosing a version of the manuscript with tracked changes and a "clean" version without tracked changes. Line numbers and Figure numbers below refer to the new clean version.

Overall evaluation

The manuscript investigates return fixations in scene viewing and visual search across tasks and species. Results are interpreted from the perspective of exploration vs. exploitation. In the modeling part, an activation-map based framework is proposed which uses concepts of attention, memory decay, and inhibition of return. While implemented as a numerical model, the current modeling work is on a qualitative level to demonstrate basic properties of return fixations. I am in favor of the manuscript which clearly adds to the literature, the innovation, however, is rather limited when compared to previous experimental as well as modeling work. Critically, the reader might want to see more analysis of data and model as well as statistical model inference in a computational biology journal.

R3.1. Abstract: The most important topic of the abstract, return fixations, has been studied intensely before, across series of experiments. What should be highlighted in the abstract are the new aspects, i.e., the experiments across tasks and species. Also, the computational model rather preliminary in its current form and some aspects lag behind already published work.

We thank the reviewer for these comments. We changed the abstract to highlight the experiments across tasks and species and the novel aspects of the model, as suggested.

R3.2. p. 2: Attention and IOR maps: Dynamical activation-based maps have been proposed as a biologically-inspired modeling framework before (SceneWalk model), published first by Engbert et al. (2015, Journal Vision), with likelihood-based parameter inference by Schütt et al. (2017, Psychological Review), and most recently in an advanced version with attentional processes around the time of saccade and fully Bayesian inference by Schwetlick et al. (2020, Communications Biology). I understand that there are differences between theses previous works and the current work, however, these previous papers should be discussed and compare with the current model. Also, I feel that a numerically implemented model w/o statistical parameter inference might be difficult to publish in a computational biology journal.

We thank the reviewer for pointing out these interesting studies, which are now cited in the text [lines 63, 64, 253]. To the best of our understanding, as the reviewer points out, these studies are quite different from our current work (see also discussion in R3.3 and R3.8). The key difference is that these manuscripts do not study or focus on return fixations, the main theme of the current manuscript.

We are excited about the prospect of building quantitative comparisons between different models of eye movements. The previous studies cited here do not constitute image-computable models that can be directly compared to all the current data here. For example, during visual search, similarity to the target plays a critical role to guide eye movements, but this is not incorporated in the above studies. Of note, we are making all the code and data publicly available and we would be very happy to see (and help develop) detailed quantitative comparisons between different models, on the same data, matching the number of free parameters per model. Such comparisons are beyond the scope of the current work but we think that they can be very important to the field.

With respect to parameter inference, the model proposed here deliberately avoids fitting data. Fitting parameters can certainly make the quantitative agreement better and we think that this is very important in building models. However, the key goal here is to develop an image-computable model with a "ventral visual cortex" image feature extractor including top-down signals plus four key ingredients; we show that this model can qualitatively capture the fundamental properties of return fixations across multiple tasks and species, without the need to retrain the model or fit parameters for each task, species or condition. We emphasize this point in lines [550-555].

R3.3. p. 2: Exploration vs. exploitation: The author should make clear that the paper by Malem-Shinitski et al. [11] tried to build a model within the same framework.

We have cited this interesting work. It is worth noting that what the authors refer to as “local attention” (exploitation), is not exactly the same fixations that we study here, i.e., return fixations. The two concepts are related: a return fixation that is close in space to a former fixation is local (e.g., saccade from fixation 3 to 4 in **Fig. 1D**). However, there could be a return fixation that is “global” (perhaps saccade from fixation 8 to 9 in **Fig. 1B** depending on the threshold to separate local versus global).
We also note that we have removed the discussion of exploration versus exploitation from the manuscript.

R3.4. p.2: In the last paragraph, the author discuss the new aspects of the manuscript, related to generalizations across tasks and species. I recommend to extend this part throughout the manuscript, but I have questions related to data quality and number of experimental subjects below.
We extended the discussion, as suggested [abstract, lines 74-93, 588-599].

R3.5. Results: A general comment on the Results is that little citations are given here; the author condensed almost all references to the introduction, however, in such a format, the details of the previous studies cannot be related to the specific results discussed here. So, while I am aware that Results is not Discussion, the reader needs more comparisons to previous work in the Results as well to appreciate the findings.
This is perhaps more of a stylistic discussion, but we generally prefer to focus on citations in the Discussion where the results can be interpreted in the context of the previous literature rather than the results. However, we appreciate the comment and the rationale proposed here by the reviewer. We have added some citations to the Results section, while still keeping most of the citations to the Discussion section.

R3.6. p. 3: Return fixations are ubiquitous: The basic finding reported in this part is not new, which should be explained to the reader. The comparison between species and tasks should be addressed more deeply.
We are not sure which reference(s) the reviewer is referring to here. We did cite previous work on return fixations and we are certainly not claiming that this is the first report of return fixations. There is a whole paragraph in the introduction [lines 53-61] citing 21 papers about return fixations. To the best of our knowledge, there are several properties of return fixations that have not been quantified before, such as return fixations in dynamic self-referenced videos. The main point that we are trying to emphasize is that return fixations occur in many tasks, species, and conditions whereas the majority of previous studies focused on a single species/task/condition. This universality is important because we seek general mechanisms that govern return fixations, as opposed to, say, only mechanisms that relate to free viewing of specific types of images, or only mechanisms involved in visual search in one particular experimental condition.

R3.7. p. 5: Subjects promptly revisit return fixations: I am not convinced about the interindividual differences results, which should be addressed in a statistically adequate way (e.g., linear-mixed effects modeling with task and subject as random factors to provide statistical evidence for stability of individual viewing behaviors).
To better reflect the consistency in viewing behavior *across* individuals, we now report our results at the subject level, as requested by Reviewer 1. We added new figures for the new analyses (**Fig. 3**, **4**, **6**, **S1**, **S3**, **S22**). We added a new section about the statistical analyses in the Methods section [1097-1104]. For example, in **Fig. 3A-H**, we ask whether the proportion of return fixations across the distribution of subjects is different than expected by the null model, rejecting the null hypothesis.
On another note, the reviewer tangentially raises an interesting point about the `stability of individual viewing behaviors`. We interpret this question to ask whether a subject would pursue the same sequence of eye movements upon encountering the same image a second time. We usually refer to this question as *within-subject* consistency, as opposed to

between-subject consistency, documented in **Fig. S4** and **S5**. Studying within-subject consistency is a bit tricky because there is a potential confound of memory (in the extreme case, if a subject is presented with the same image two trials in a row, their pattern of eye movements may be different, not because of a lack of consistency, but because of a strong memory for the previous viewing). We did not study within-subject consistency in the current work because in most cases images were presented only once. In previous work on visual search, we extensively characterized within-subject consistency and compared it to the between-subject consistency (Zhang et al, 2018).

R3.8. p. 5: The authors state that there was "no consistent relationship between the duration of the to-be-revisited fixation and non-return fixation." One would see a bit more details in this direction. For example, Schwetlick et al. (2020, Communications Biology, Fig. 5 + 6) demonstrate that there is systematic relation between turn angles and saccade amplitude as well as fixation durations. This has implications for the modeling part as well (see my next point).

We thank the reviewer for pointing us to the work of Schwetlick *et al* 2020, which we now cite. We added a new supplementary figure (**Fig. S23**) in which we show the consistent relation between turn angles and saccade amplitudes, confirming the trend previously shown by Schwetlick et al 2020.

It should be noted that Schwetlick *et al* did not study return fixations. The work considers "reverse saccades", which are cases where there is a reversal in the saccade motion direction. As can be appreciated in **Fig. 5A**, most return fixations are not reverse saccades (depending on the exact threshold, all the return fixations that are not in the last bin (180 degrees) or all the return fixations between 0 and 90 degrees could be considered in this category). Conversely, the majority reverse saccades are not return fixations (again, depending on the exact threshold, all the non-return fixations in the 180 degree bin or those between 90 and 180 degrees in **Fig. 5B** would belong in this category. Note that **Fig. 5** shows percentages; there are more non-return fixations than return fixations (**Fig. 3A-H**); therefore, most reverse saccades are not return fixations). And the same applies to the "forward saccades" described in Schwetlick et al, which are different from our non-return fixations. In other words, although there is some overlap, Schwetlick *et al* largely do not analyze the same fixations that we study here.

We further discuss turning angles and the model in the following point.

R3.9. p. 7: A computational model: Because of the complex coupling between fixation durations and saccade amplitudes to saccadic turning angle, the proposed model should be investigated with respect to such statistics. Even if the author would like to stay at a qualitative level (which is OK with me), a model that cannot reproduced basic statistics such as the distribution of turning angle would not be acceptable when it is used as a qualitative tool to explain return fixations.

We thank the reviewer for this suggestion. We introduced several new figures (**Fig. 5, Fig. 9, Fig. S7, Fig. S15, Fig. S19**) in which we show the distribution of turning angle of fixations for the experimental data and also for the model, as suggested. We also discuss the turning angles in the text, [lines 243-256, 423-425, 441, 450-451].

Briefly, we observed two main trends depending on whether the fixation returned to a previously visited location or not. For return fixations, the turning angle distributions peaked at 180 deg in all tasks and species (**Fig. 5A**). This trend changed when considering non-return fixations which followed a U-shaped distribution with slight peaks at 0 and 180 degrees, with a bias toward 0 deg in humans and a bias toward 180 deg in monkeys (**Fig. 5B**). These turning angle biases have been previously shown in Schwetlick et al. 2020, as pointed out by the reviewer. We cite and discuss this work now in the main text.

Our model was able to qualitatively capture these trends (**Fig. 9B-C**). For return fixations, the model showed a consistent peak at 180 deg, similar to the experimental observations. For non-return fixations, the model tended to show a more uniform distribution with slight

preference toward small angles in tasks 1-4, as in humans, and a slight preference toward large turning angles in tasks 5-6, as in monkeys.
The reviewer also briefly mentions fixation durations in the question. The proposed model does not really have fixation durations and therefore we cannot evaluate the observations in **Fig. 4A** in the model. We clarify this now in the text [lines 412-413, 589-591].

R3.10. p. 8: The assumption that of constant acuity over the entire visual field is hard to accept in a biologically inspired model (see also last para of the Discussion on p. 11). Other papers (e.g., on the SceneWalk model) try to implement limited attention by a Gaussian attentional aperture, which naturally explains the saccade-size distributions. The arbitrary assumption of a fixed Gamma-type distributed should be avoided. Also, the author should visualize the saccade-size distribution of the model in comparison to the experimental data. I think the idea that oculomotor limitations [51, 52] provide the answer to this question is experimentally falsified, at least this is no longer a theoretically motivated argument.
There are several important points in this question. First, indeed, eccentricity-dependent sampling is an important aspect of primate vision and we emphasize in the discussion that this is a limitation of the current model [579-582]. As the reviewer points out, several studies have built models with eccentricity-dependent sampling .
As suggested, we now show the saccade size distributions both for the experimental data (new **Fig. S6A**), as well as for the model (new **Fig. S6B**). The saccade size distributions are very similar to those reported in earlier studies, including **Fig. 5C** in Engbert et al 2015. These distributions resemble a gamma distribution.
It is important to note that we argue that it is important to have a constraint on the size of saccades. Empirically, we observe that subjects do *not* make very large saccades (**Fig. S6A**, see also Zhang *et al*. 2018 and Carpenter 1988). The current model is agnostic as to *why* subjects do not make large saccades. While we suspect that eccentricity-dependent sampling contributes to this constraint, we also suspect that it is not the only contribution (see the excellent treatise by Carpenter 1988).
Consider a highly salient object in the far periphery (say 60 degrees): eccentricity-dependent sampling may "blur" this object but the location can still be highly salient. However, subjects do not often make such large saccades. Instead, they rotate their head. And if they cannot rotate their head (e.g., in head-fixed experiments), they will make multiple smaller saccades (e.g., 3x20 instead of 1x60). If really forced to make a large saccade, subjects usually still make an approximation followed by small corrective saccades.
Why is it that subjects don't make such a large saccade? We suspect that the eyes are not built or trained to make such large saccades (Carpenter 1988). We would appreciate it if the reviewer could provide a reference for work that falsifies the notion that such oculomotor limitations constrain the size of saccades.
Independently of the discussion of the origin of this constraint, the model presented here does not depend on where the saccade size constraint comes from, in the same way that the model proposes a memory module without explicitly stating where this memory comes from. Of course, all of this is not to say that eccentricity-dependent sampling is not important. We agree with the reviewer that it clearly is important, but we suspect that it is unlikely to be the only constraint on large saccades.

R3.11. p. 10: "… has very few parameters": I agree, but this is precisely the reason why one would believe that statistical parameter inference is possible and should be investigated. Important questions are related to this point, e.g., the question whether the model could explain interindividual differences via parameter variation.
This is also an interesting direction, and we point this out in the discussion [538-555]. There are many potential parameters that could be fine-tuned, including the actual weights in the "visual cortex" module and the specific layers selected, both of which are critical for the calculation of saliency (there is extensive work on models of bottom-up saliency dating back

to Itti and Koch 2000, if not earlier), which layers are used to provide top-down modulation in search tasks (see Zhang *et al* 2018 and Gupta *et al* 2021 for discussion of this point), the memory decay function (**Fig. S10A**), the saccade size prior maps (**Fig. S10B**), the threshold to define return fixations (**Fig. S22**), and the relative contributions of the different modules. Fine-tuning all of these components can only lead to better fits for a specific species, a specific task, or a specific condition. In addition, although subjects tended to be quite consistent with each other (e.g., **Fig. S4**, **S5**), as the reviewer points out, it is also likely that fine-tuning parameters would lead to better fits for individual subjects. With 9 main figures and 23 supplementary figures, we think that these questions are beyond the scope of the main goal here, which is to build a proof-of-principle generalistic computational model that can capture return fixations and their properties across tasks and species. We agree with the reviewer that model parameters can be tuned to get better fits and point this out in the discussion [lines 548-550].

R3.12. p. 11: "return fixations showed longer fixation durations". See also Rothkegel et al. (2019, Scientific Reports, Fig. 7C). The bottom line from my comments is that the reader needs more details/visualizations/analyses on the data and model output to understand the results.

We thank the reviewer for the reference to Rothkegel *et al*, which we now cite. We are not sure whether there was a specific question here about return fixations. We introduced 17 new figures to better describe the data and the output of the model (**Figs. 3, 4, 5, 8, 9, S1, S3, S6, S7, S15, S16-S20, S22, S23**).

R3.13. Methods: The presentation times were quite different across the different experiments. I would be surprised if this does not have an impact on the results. Please comment based on additional analyses.

We agree with the reviewer's point. One specific concern that we had is that the pattern of fixations may change over the course of a trial. For example, and only as a trivial thought experiment, consider an experiment where a subject is forced to free view the same image for 10 hours. It is very likely that every fixation will become a return fixation eventually. Of course, our durations are only several seconds, but this thought experiment illustrates how the presentation times could impact the results as the reviewer intuits.

To address this question, we reanalyzed the data by focusing exclusively on the first 6 fixations for all experiments (**Fig. S3**). The number 6 was chosen because it was approximately the minimal number of fixations per trial across all the experiments. The results show that the proportion of return fixations still exceeds chance levels across the different experiments in this case.

R3.14. p. 13: The use of a 30 Hz eye tracker with low spatial resolution is possible, however, role of smaller saccades will not be addressed in this case. Here, the main sequence and overall saccade amplitude distribution should be plotted and investigated to emphasize the reliability of the results.

The sampling rates were: 500 Hz (**Fig. 2A-D**), 1000 Hz (**Fig. 2E**), 120 Hz (**Fig. 2F**), 30 Hz (**Fig. 2G-H**). In **Fig. 2A-D**, we estimated the error in eye tracking to be below 1 dva. In **Fig. 2E-F**, we estimated the error in the eye tracking to be below 0.5 dva, and in **Fig. 2G-H**, we estimated the error in the eye tracking to be below 1 dva. As the reviewer points out, the video data are more difficult to interpret. We have a whole section in the text to describe how we analyzed fixations in the video data [lines 683-709], see also **Fig. S2**.

As suggested, we also plotted the distribution of saccade sizes for all of the experiments in new **Fig. S6A**.

Additionally, we added **Fig. S22**, showing the effect of the threshold to define return fixations on the results. Although, as expected, the proportion of return fixations increases when the threshold increases from 1 to 4 dva, the overall conclusions do not change. In fact, the

model provides a slightly better account of the data when using a larger threshold than the one used throughout the text (**Fig. S22,** [lines 1014-1024]).

R3.15. p. 16: The Gaussian in Eq. (4) needs a negative exponent.
We corrected this. Thanks!

Typos:
- “occulomotor” throughout the manuscript
We corrected this. Thanks!
