# Supplementary material for: Look twice: A generalist computational model predicts return fixations across tasks and species
Source: PLoS Comput Biol. 2022 Nov 22;18(11):e1010654. doi: 10.1371/journal.pcbi.1010654 (PMC9681066; doi:10.1371/journal.pcbi.1010654)
Supplement: Attachment — Submitted filename: CompBio_ReviewPointByPoint_Round2.pdf [file pcbi.1010654.s026.pdf]

Point By Point Responses

Text in small black font shows the reviewers' questions. Text in blue indicates our responses. We are enclosing a version of the manuscript with tracked changes and a version without tracked changes. Line numbers and figure numbers below refer to the version without tracked changes.

Reviewer #1:

R1.1 As indicated above, I am satisfied by the responses provided by the authors, as most of my comments were focused on considerations of aspects of the tasks or model that might be worth addressing in the introduction or discussion. These were addressed, and the "ablation" approach does address the relatively importance of the different modules. If there were one thing I think might still be missing in the general discussion of the model is the simplicity with which space is being treated. This makes sense for CNNs, where all the images can be magically aligned and the WTA operation is simple enough. Perhaps some comment about how this simplification has only the vaguest support from existing neuroscientific studies, as these maps appear to exist in some forms, but they are spread across different brain areas and the registration of space is hardly a solved problem.

We agree with the reviewer that CNN preserves spatial locations when extracting visual features, and that makes it easy to combine attention maps and use the winner-take-all mechanism based on the predicated attention maps. We have added this point in the discussion section (lines 505-510).

R1.2 While I think the paper make a valuable contribution and appreciate the effort put into the revision, I do have a couple of remaining issues/questions.

1. The authors suggest that they have set aside the explore/exploit viewpoint, but the idea is still introduced in 59ff and it's not really tied to anything that follows.

We had kept this line to cover relevant background rather than the focus of our paper. We have rewritten that line to avoid confusion.

R1.3 The ablation approach and the conclusions are highlighting on page 10, but all of the figures are buried in the supplementals. I'd think it might be worth somehow creating some figure that highlights the take home message from these and then referring to the supplemental data figures for the nitty gritty details?

As suggested, we have added Table 1 in the main text, summarizing the key results for the ablated models and we refer to the supplementary figure for the details.

R1.4 For the Null model, it is a little awkward that this couldn't be framed as a totally ablated model or put into this framework. I see the argument presented in 932ff, but it would seem more parsimonious to connect these.

We introduced several versions of the model ablating different components in Figures S16-S20 and Table 1. We are not sure what the reviewer refers to as a "totally ablated model". An extreme interpretation of "total ablation" would lead to a model that selects the maximum activation and stays at that location forever, which does not seem like a fair null comparison. To avoid this problem, we introduced a random component so that the null model could explore different locations. Such a null model tends to make larger saccades. We were concerned that such larger saccades would lead to concluding that our model provides a better description of the data. Hence, we included the saccade size constraint into the random null model. If we were to remove this constraint, none of the conclusions in the paper would change, the actual model would appear to be even better in comparison to the null. We consider this to be a stricter null hypothesis and a higher bar for our model to pass than a totally ablated model.

R1.5. There is also no main figure for the consistency analysis (181ff)?

As suggested, we have now added two new panels in Figure 1 to illustrate the between-subject consistency in return fixations on two example images.

R1.6 180 degree turns. It's not clear how this metric is not confounded by the "offset" measure. I still don't understand how a 1 offset return saccade doesn't, by definition, require a 180-turn?

The reviewer is interpreting the results correctly. Indeed, a 1-offset return fixation *requires* a 180-degree turn. However, a 180-degree turn does *not* necessarily lead to a return fixation. As a coarse analogy, consider driving from New York to Baltimore, making a 180-degree turn and driving to Philadelphia (or continuing past New York to Boston). There is no return to New York in these

cases and neither case would not be considered a return fixation even though both involve a 180-degree turn. Thus, 1-offset return fixations imply 180-degree turns but the reverse is not true.

From Figure 3J, we observed that the distribution of return offsets in all experiments showed rapid decay. Most of the return fixations had an offset of 1. Consistently, we observed that the distribution of turning angles peaked at 180 degrees (Figure 5A and S7A) .

R1.7 Between subject consistency (1079ff): For the entropy metric are there enough data to make sure this measure can be made reliably? It seems like there will be many cells in the grid with very few points?

The reviewer rightly points out the sparsity of the fixation data. We explain in lines 1110-1114 how this was addressed in the entropy calculation.

R1.8 Figure 4: Return fixations tend to follow smaller saccades and last longer. First, given the figure caption, A and B (or the caption) could be reversed.

As suggested, we have now reversed the order of the caption.

R1.9 But more critically, the duration argument is really driven by the search tasks, and it's not clear if the returns are to the to be found target so that the duration is more a function of the recognition part of the task and not really a "universal" property of return saccades?

We thank the reviewer for pointing this out and we agree with this point. We now emphasize in the text that the longer durations are predominantly observed in the search tasks (lines 231-238).

Additionally, we have extended Figure 4 to separately analyze fixation durations for return, to-be-revisited, and non-return fixations at target and non-target locations in the three visual search tasks (Figure S24, lines 249-254). The return fixation durations are longer at target locations than at non-target locations, implying that object recognition might be important for return fixations as the reviewer points out.
